# Decrease in gamma-band activity tracks sequence learning

**DOI:** 10.3389/fnsys.2014.00222

**Published:** 2015-01-21

**Authors:** Radhika Madhavan, Daniel Millman, Hanlin Tang, Nathan E. Crone, Fredrick A. Lenz, Travis S. Tierney, Joseph R. Madsen, Gabriel Kreiman, William S. Anderson

**Affiliations:** ^1^Boston Children's Hospital, Harvard Medical SchoolBoston, MA, USA; ^2^Program in Neuroscience, Harvard UniversityCambridge, MA, USA; ^3^Program in Biophysics, Harvard UniversityCambridge, MA, USA; ^4^Department of Neurology, Johns Hopkins School of MedicineBaltimore, MD, USA; ^5^Department of Neurosurgery, Johns Hopkins School of MedicineBaltimore, MD, USA; ^6^Department of Neurosurgery, Brigham and Women's HospitalBoston, MA, USA; ^7^Department of Neurosurgery, Boston Children's Hospital, Harvard Medical SchoolBoston, MA, USA

**Keywords:** sequence learning, memory, intracranial recordings, field potentials, gamma frequency oscillations, human neurophysiology

## Abstract

Learning novel sequences constitutes an example of declarative memory formation, involving conscious recall of temporal events. Performance in sequence learning tasks improves with repetition and involves forming temporal associations over scales of seconds to minutes. To further understand the neural circuits underlying declarative sequence learning over trials, we tracked changes in intracranial field potentials (IFPs) recorded from 1142 electrodes implanted throughout temporal and frontal cortical areas in 14 human subjects, while they learned the temporal-order of multiple sequences of images over trials through repeated recall. We observed an increase in power in the gamma frequency band (30–100 Hz) in the recall phase, particularly in areas within the temporal lobe including the parahippocampal gyrus. The degree of this gamma power enhancement decreased over trials with improved sequence recall. Modulation of gamma power was directly correlated with the improvement in recall performance. When presenting new sequences, gamma power was reset to high values and decreased again after learning. These observations suggest that signals in the gamma frequency band may play a more prominent role during the early steps of the learning process rather than during the maintenance of memory traces.

## Introduction

Memory formation takes place over multiple time scales ranging from seconds to days. Declarative memory involves several different processes: the initial storage, or encoding, of information; the subsequent reactivation, or retrieval, of this memory trace; and, over time, a process of consolidation, through which the initially transient memory trace is converted into a longer lasting form. Lesion, behavioral and neurophysiological studies suggest that there exist different brain structures and circuit mechanisms associated with these different temporal scales, including single-instance iconic memory (Coltheart, 1980), short-term memory lasting for seconds (Baddeley, 1992) and long-term memory that persists over hours to multiple days (Morris et al., 1982; Squire and Zola-Morgan, 1991; Bliss and Collingridge, 1993).

The amount of training and exposure required to learn or acquire information varies significantly across different tasks. At one extreme, single events can lead to lasting episodic memories (Tulving, 2002). At the other end of the spectrum, implicit acquisition of skills or certain forms of perceptual learning are characterized by gradual improvement over thousands of repetitions and prolonged periods of time (Salmon and Butters, 1995; Knowlton et al., 1996). Less is known about the intermediate regime whereby learning takes place over the course of a few repetitions and several seconds to minutes such as learning a new phone number or a new navigational route.

Here we investigate the neural signals underlying declarative sequence learning through recall, over time scales of minutes in a temporal-order recall task. We focus our study on intracranial field potential (IFP) signals in the gamma frequency band from 30 to 100 Hz. Field potential activity in this frequency range is correlated with the underlying spiking activity (Ray et al., 2008). Additionally, several studies have documented significant modulation in gamma frequency band power during short-term (Pesaran et al., 2002; Axmacher et al., 2007; Jutras and Buffalo, 2010) and long-term memory (Montgomery and Buzsáki, 2007) in rodents, monkeys and humans. Sustained increase in the gamma frequency band power has been observed during maintenance of working memory in monkeys (Pesaran et al., 2002) and humans (Tallon-Baudry et al., 1999; Howard et al., 2003). Gamma band activity has also been shown to reflect successful retrieval of long-term memory in humans (Sederberg et al., 2003). Further, given the high frequency of gamma oscillations, gamma band activity has been thought to mediate coincidence detection and synaptic plasticity (Jutras and Buffalo, 2010). Previous work evaluating the role of gamma frequency band oscillations in humans has focused on examining learning and memory formation in multiple tasks after averaging responses to single presentations of stimuli (for a review, see Jensen et al., 2007). We extend these efforts by quantifying the modulation in the gamma frequency band as a function of the behavioral improvement over trials during declarative learning of temporal sequences. We hypothesized that the behavioral changes in recall performance over trials would be reflected in the trial-by-trial modulation of activity in the gamma frequency band.

We recorded IFPs from 1142 subdural electrodes implanted in 14 epileptic patients while they performed one of two sequence recall tasks. To examine neural activity during the acquisition of new sequences, we evaluated the trial-by-trial changes in gamma-band power while quantifying the subjects' behavioral improvements in explicit recall of item order. In each trial, there was an increased gamma power during the recall phase with respect to baseline. In those subjects that were able to successfully learn the sequences, we observed a reduction in the degree of gamma-band power enhancement over trials during sequence *recall* accompanying behavioral improvement. This inverse correlation between sequence-order recall performance and power in the gamma-band was present in the temporal lobe, most prominently in the parahippocampal and middle temporal gyri. Changes in gamma-band power were absent in cases where the learning performance criterion was not reached. These observations suggest that gamma-band activity may take a prominent role during the initial process of learning and memory retrieval and a lesser role once learning has been established.

## Materials and methods

### Patients

**Figure 1:**
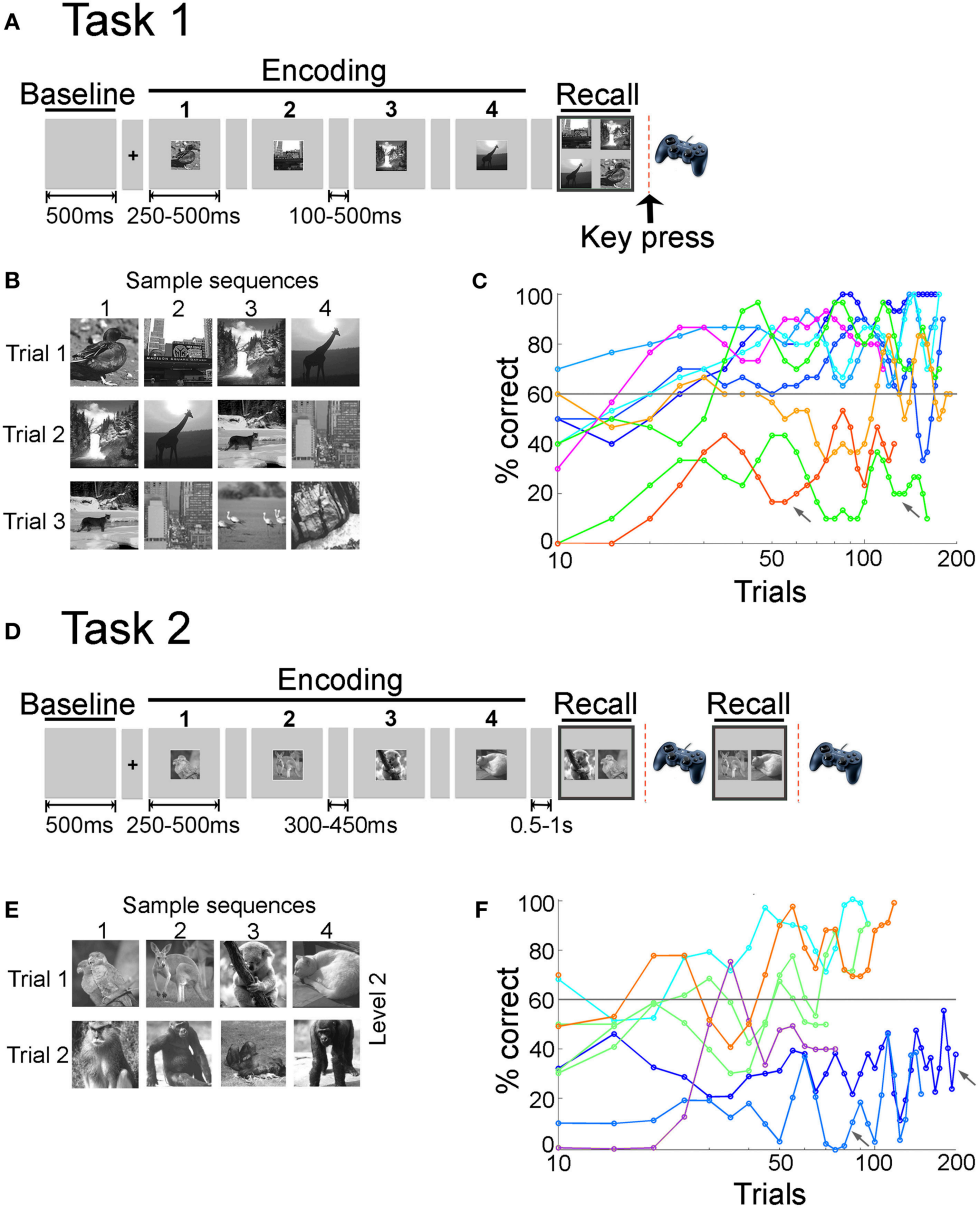
**Sequence encoding tasks and learning behavior. (A)** In Task 1, each trial started with a fixation screen (100 or 500 ms) followed by a sequence of four images, each one presented for either 250 or 500 ms, referred to as the encoding phase. After a short delay (100 or 500 ms), the four images were presented together on the screen in the recall phase. The subjects had to report the order of the four images using a gamepad (key press) (Materials and Methods). **(B)** Sample images presented during Task 1. Each sequence consisted of four images from a pool of eight images per session. Images were always presented in the same order and three or five overlapping sequences were shown per session. **(C)** Behavioral performance for Task 1. Performance in a given trial was considered correct if the subject reported the right order for *all* four images in the sequence (out of the 24 possible combinations in which four buttons can be pressed, only one of them was correct). Here we show the average performance (percentage of correct trials, y-axis) for each subject (eight subjects) and session in bins of 10 trials (x-axis, note the logarithmic scale). Colors indicate different subjects; for one subject there are two separate lines, indicating different sessions (green line). The gray line indicates the learning criterion of 60% correct (Materials and Methods). Arrows indicate sessions in which the subjects' performance did not satisfy the learning criterion. On average, subjects needed 28 ± 11 [mean ± standard deviation (SD)] trials to reach the learning criterion in Task 1. **(D)** In Task 2, each trial started with a fixation screen (300 or 450 ms) followed by a sequence of 2, 4, 6, or 8 images (levels 1–4 respectively, Materials and Methods). During the recall phase, two of the images were shown and subjects had to indicate which image appeared earlier in the sequence. For sequence lengths >2, recall was sequentially tested for two pairs of images randomly chosen from all possible image pairs in the sequence. **(E)** Sample images presented during Task 2 (here, level 2, length = 4). Images were always presented in the same order and five or six non-overlapping sequences were shown per level. Each level consisted of different images. **(F)** Behavioral performance for Task 2. Performance in a given trial was considered correct if the subject reported the correct order for *all* pairs presented in the recall phase. Here we show the average performance (percentage of correct trials, y-axis) for each subject (six subjects) and level in bins of 10 trials (x-axis, note the logarithmic scale). Colors indicate different subjects; for one subject there are two separate lines, indicating different levels (green line). While subjects participated in more than one level, only levels with more than 30 trials are included in this graph. Arrows indicate levels in which the subjects' performance did not satisfy the learning criterion. On average, subjects needed 25 ± 22 trials to reach the learning criterion in Task 2.

We analyzed data from 14 patients with drug-resistant epilepsy (9 male, 12 right handed, 13–42 years old; one of the 14 patients was a child, aged 13) undergoing invasive monitoring as part of the procedure to determine the seizure focus for potential surgical resection. Each patient participated in one of two versions of the sequence recall task (Figure 1); eight patients participated in Task 1 and six in Task 2. Each patient participated in one testing session (7 subjects in Task 1, 6 subjects in Task 2) or two testing sessions (1 subject in Task 1). The research protocol was approved by the institutional review boards at the Boston Children's Hospital (BCH, Boston, MA), the Johns Hopkins Hospital (JHH, Baltimore, MD) and Brigham and Women's Hospital (BWH, Boston, MA). Informed consent was obtained from the patients.

### Electrophysiology

Subjects were implanted with multiple intracranial subdural electrodes (range = 34–126 electrodes). Electrode locations were determined by the clinical team to identify seizure foci to guide potential subsequent surgery. Because the clinical procedure of identifying seizure foci involves placing electrodes in any region that is potentially epileptogenic, the majority of the recordings are from brain regions outside the seizure focus area. Patients underwent a craniotomy for subdural implantation of electrode grid and strip electrodes followed by 1–2 weeks of continuous monitoring. Intracranial electrode grids/strips were 2 mm in diameter (with 1 cm separation, Adtech, Racine, WI) placed directly on the surface of the cortex. The signal from each electrode was amplified and filtered (0.1–100 Hz) with a sampling rate of 256 or 500 Hz at BCH and BWH (XLTEK, Oakville, ON, Canada) and 1 kHz at JHH (Stellate, Natus Medical Inc., San Carlos, CA). A notch filter was applied at 60 Hz to remove line noise artifacts (4th order bandstop butterworth filter between 58 and 62 Hz implemented in MATLAB's *butter* function). The recordings from each electrode were re-referenced to the common average across all electrodes in the same subject to remove common noise shared across electrodes. In order to remove potential movement artifacts, we computed the distribution of the field potential power in all trials for each electrode. We excluded from further analysis those trials where the power was more than three standard deviations from the mean.

### Electrode localizations

Electrode locations were computed by co-registering the preoperative magnetic resonance imaging with the post-operative computed tomography scans. For each subject, the 3D brain surface was reconstructed and then an automatic parcellation was performed using Freesurfer (Destrieux et al., 2010). The electrode positions were mapped onto 74 brain areas (Destrieux et al., 2010); these are listed together with the Talairach coordinates in the main text and in Table 1. Based on these coordinates, the electrodes were superimposed on the reconstructed brain surface for visualization purposes.

**Table 1:** **Location of all electrodes analyzed in this study (*N* = 14 subjects) conveying the range of sampled locations**.

**Area**	***n***	**Median Talairach**		
Middle temporal gyrus (MTG)	116	−63.45	−22.50	−15.60
Superior temporal gyrus (STG)	97	−63.70	−11.90	−3.50
Inferior temporal gyrus (ITG)	92	−48.05	−29.60	−23.55
Temporal pole (T-pole)	55	−22.80	4.60	−33.80
Supramarginal part of the inferior parietal gyrus (G-P-Inf_Supramarginal)	50	−62.90	−34.35	31.40
Postcentral gyrus (G-postcentral)	49	−52.70	−20.70	46.40
Precentral gyrus (G-precentral)	49	−54.80	1.90	36.40
Parahippocampal gyrus (PHG)	47	−19.10	−16.30	−30.20
Middle frontal gyrus (MFG)	45	−32.90	36.70	24.60
Orbital gyrus (G-orbital)	35	14.20	42.30	−21.00
Fusiform gyrus (G-Fusiform)	30	−33.35	−45.30	−18.05
Angular part of the inferior parietal gyrus (G-P-Inf-Angular)	29	−46.90	−67.10	29.70
Opercular part of inferior frontal gyrus (G-F-Inf-Opercular)	28	−56.15	16.00	5.05
Middle occipital gyrus (MOG)	28	−39.30	−85.10	13.70
Subcentral gyrus (central operculum) and sulci (G-S-Subcentral)	27	−61.70	−7.20	11.40
Triangular part of the inferior frontal gyrus (G-F-Inf-Triang)	27	−52.00	28.60	−3.20
Superior parietal gyrus (SPG)	25	−27.90	−50.80	57.40
Inferior occipital gyrus and sulcus (G-S-Inf-Occipital)	14	−46.50	−79.00	−8.15
Precuneus (G-precuneus)	14	1.30	−51.10	35.95
Occipital pole (O-pole)	13	1.70	−95.20	−3.30
Superior temporal sulcus	12	−50.20	−64.75	8.50
Cuneus	9	1.00	−77.50	17.40
Inferior frontal sulcus	9	−52.30	30.10	11.80
Postcentral sulcus	9	34.20	−34.30	49.50
Superior frontal gyrus	8	−8.80	35.80	54.65
Superior occipital gyrus	8	−16.95	−82.40	30.60
Planum temporale or temporal plane of the superior temporal gyrus	7	−63.90	−44.50	18.50
Inferior temporal sulcus	7	−53.00	−46.60	−21.10
Frontomarginal gyrus (of Wernicke) and sulcus	6	20.40	65.05	−20.05
Lingual gyrus	6	−9.80	−55.15	−8.90
Superior frontal sulcus	5	20.10	46.00	42.40
Middle occipital sulcus and lunatus sulcus	5	−28.30	−88.80	8.90
Transverse frontopolar gyri and sulci	4	−20.70	45.50	−21.80
Orbital part of the inferior frontal gyrus	4	49.65	24.70	−11.00
Gyrus rectus	4	1.85	26.10	−27.90
Planum polare of the superior temporal gyrus	4	−47.25	10.65	−14.55
Central sulcus	4	38.35	−7.50	46.40
Intraparietal sulcus and transverse parietal sulci	4	−3.05	−53.40	45.45
Anterior occipital sulcus	4	45.45	−76.05	−3.75
Posterior dorsal cingulate gyrus	3	0.80	−26.40	37.70
Anterior transverse collateral sulcus	3	−38.50	−26.90	−23.40
Middle frontal sulcus	3	29.10	47.10	27.80
Orbital sulci (H-shaped sulci)	3	−17.00	32.80	−24.90
Paracentral gyrus and sulci	2	0.45	−28.10	47.75
Inferior part of the precentral sulcus	2	1.20	16.20	38.10
Subparietal sulcus	2	−0.15	−39.95	32.75
Anterior transverse temporal gyrus (of Heschl)	1	−58.00	−26.30	1.80
Temporal plane of the superior temporal gyrus	1	−63.70	−4.10	16.70
Sulcus intermedius primus (of Jensen)	1	−55.60	−58.80	33.10
Occipital temporal lateral sulcus	1	−48.60	−47.60	−26.30
Lingual sulcus	1	−68.60	−45.30	−0.10
Lateral orbital sulcus	1	−24.50	65.80	−14.00
Medial orbital sulcus (olfactory sulcus)	1	11.10	28.50	−26.70
Superior segment of the circular sulcus of the insula	1	−63.70	−4.10	16.70
Parieto-occipital sulcus (or fissure)	1	1.70	−66.90	32.30
Superior part of the precentral sulcus	1	34.80	3.80	57.00
Total number of electrodes assigned cortical area	1017			
Electrodes not assigned cortical area (see legend)	125			
Total	1142			

*This table lists the names and abbreviations for each location as used throughout the text and figures (for further details about each location and the corresponding anatomical landmarks, see Destrieux et al., 2010). We show the median Talairach coordinates for each region. This is provided only as a coarse measure, and should be taken as an approximation since it is not clear whether a median operation is valid in Talairach space. The total number of electrodes in this table is 1017 instead of 1142; there were 125 electrodes (11%) for which we could not obtain accurate mapping on to the brain's surface (e.g., interhemispheric strip electrodes)*.

No seizures occurred during the experiment. In the Results section, we describe the activity of 51 electrodes that showed decreased gamma band power concomitant with behavioral learning. Out of these 51 electrodes, 6 (11%) were a part of the seizure onset zone (as annotated by the clinical team). Removing these electrodes from the analyses would not alter the conclusions. For example, the modulation index (defined below) would be −0.27 ± 0.03 instead of −0.22 ± 0.03 as reported in the text.

### Stimulus presentation and task

Subjects participated in one of two sequence recall tasks. In both tasks, subjects were instructed to learn and recall the order of image sequences through repeated recall. Subjects were informed that multiple image sequences would be presented during the session, and memory for sequence order would be tested on every trial following sequence presentation. They were also informed that each individual sequence would be repeated multiple times to facilitate learning. Subjects were not encouraged to say the sequence aloud or talk during the task to avoid speech artifacts. Images were presented on a 15 inch (screen diagonal: 15.40 inch, width: 13.06 inch, height: 8.16 inch) MacbookPro laptop computer (using the MATLAB Psychophysics toolbox Brainard, 1997). Electrophysiological recordings were synchronized with behavioral events via TTL pulses.

#### Task 1

In a trial, subjects were presented with a temporal sequence consisting of four images and instructed to report the sequence order using a keypad after a short delay (Figure 1A). Multiple image sequences were presented during a recording session. All images were presented on the center of the screen. A trial started with a fixation spot (100 or 500 ms). The 500 ms time interval before fixation is referred to as the Baseline phase of the task. Images were contrast-normalized gray-scale digital photographs (256 × 256 pixels) of indoor scenes, outdoor scenes and animals. Each sequence consisted of four images from a pool of eight images per session. Images were always presented in the same order and three overlapping sequences (2 subjects) or five overlapping sequences (6 subjects) were shown per session (Figure 1B). The specific set of four images in each trial was chosen randomly within the image sequences. If the subject participated in more than one session, different images were used in each session. Sequences were presented in pseudo random order. Images were presented for 250 ms and were followed by a gray screen for 500 ms. After the first two subjects, we increased the image presentation time to 500 ms and decreased the inter-image interval to 100 ms to ensure adequate time for image viewing. The interval where the four images were sequentially presented is referred to as the Encoding phase of the task. Following a delay of the same duration as the inter-image interval, a choice screen was presented (Recall phase). The choice screen consisted of all four images in the sequence presented together on the screen. Following the presentation of the choice screen, the subject had to order the images on the choice screen in the order in which they had appeared using four buttons on a gamepad. The size of the images on the choice screen was the same as during the image presentation and the position of the individual images on the screen was randomized in each trial. Hence, even though for any given sequence the same images were presented on the choice screen, the relative positions of the images during the choice screen were not the same. This ensured that subjects recalled the sequence (image order) and not the relative position or motor mapping of the images on the choice screen. The interval between trials (time from the last key press to the fixation screen for the next trial) was 2 s. The average number of trials in each session was 170 ± 26 (mean ± SD). Psychophysics studies for Task 1 were conducted on eight healthy subjects (8 female, right-handed, 18–26 years old). No electrophysiological recordings were conducted on the healthy subjects. Four subjects participated in 1 session and 4 subjects participated in 2 sessions. In the psychophysics tests with healthy volunteers, the images were presented for 500 ms and were followed by a gray screen for 100 ms. Performance for epilepsy and non-epilepsy subjects were analyzed using the same procedures and is shown in Supplementary Figures 1a,b.

#### Task 2

We considered a variant of the task to examine learning individual sequences, different complexity levels and simplify the motor response. In a given trial, subjects were presented with a temporal sequence consisting of 2, 4, 6, or 8 images (Figure 1D). A trial started with a fixation spot (300 or 450 ms). The baseline period was defined as in Task 1. Each sequence consisted of 2, 4, 6, or 8 images referred to as Levels 1–4. Images within a given sequence were always presented in the same order and five (2 subjects) or six non-overlapping sequences (4 subjects) were shown per level (Figure 1E). Subjects sequentially participated in multiple levels with increasing numbers of images per sequence. Different images were used in each level. Sequences were presented in pseudo random order. Images were presented for 250 ms (5 subjects) or 500 ms (1 subject) and were followed by a gray screen for 300 ms (3 subjects) or 450 ms (3 subjects). Similar to Task 1, the interval where the image sequence was presented is referred to as the Encoding phase of the task. Following a delay of 1 s, a choice screen was presented (Recall phase). The choice screen consisted of a randomly chosen pair of images from the image sequence presented together on the screen. Following the presentation of the choice screen, the subject had to indicate which image had appeared earlier in the sequence using one of two buttons on a gamepad. The size of the images on the choice screen was the same as during the image presentation and the position of the individual images on the choice screen was randomized in each trial. Order recall for image pairs was tested for two random pairs of images in each trial in levels 2–4. If subjects made eight correct choices in a set of 10 trials, they proceeded to the next level with increased sequence length. If subjects could not cross this threshold within the first 50 trials they continued on the same level for the rest of the test session. Out of 6 subjects that participated in Task 2, 3 subjects participated in levels 1,2, 2 subjects participated in levels 1–3 and one subject participated in levels 1–4. The interval between trials (time from the last key press to the fixation screen for the next trial) was 2 s. The average number of trials in each level was 78 ± 58 (mean ± SD). Psychophysics studies for Task 2 were conducted on five healthy subjects (3 male, right-handed, 18–33 years old). No electrophysiological recordings were conducted on the healthy subjects. Each subject participated in one session with multiple levels. In the psychophysics tests with healthy volunteers, the images were presented for 300 ms and were followed by a gray screen for 400 ms. Performance for epilepsy and non-epilepsy subjects were analyzed using the same procedures and is shown in Supplementary Figures 1c,d. Note the difference in gender distribution between epilepsy and non-epilepsy subjects.

## Data analyses

### Learning curves

**Figure 2:**
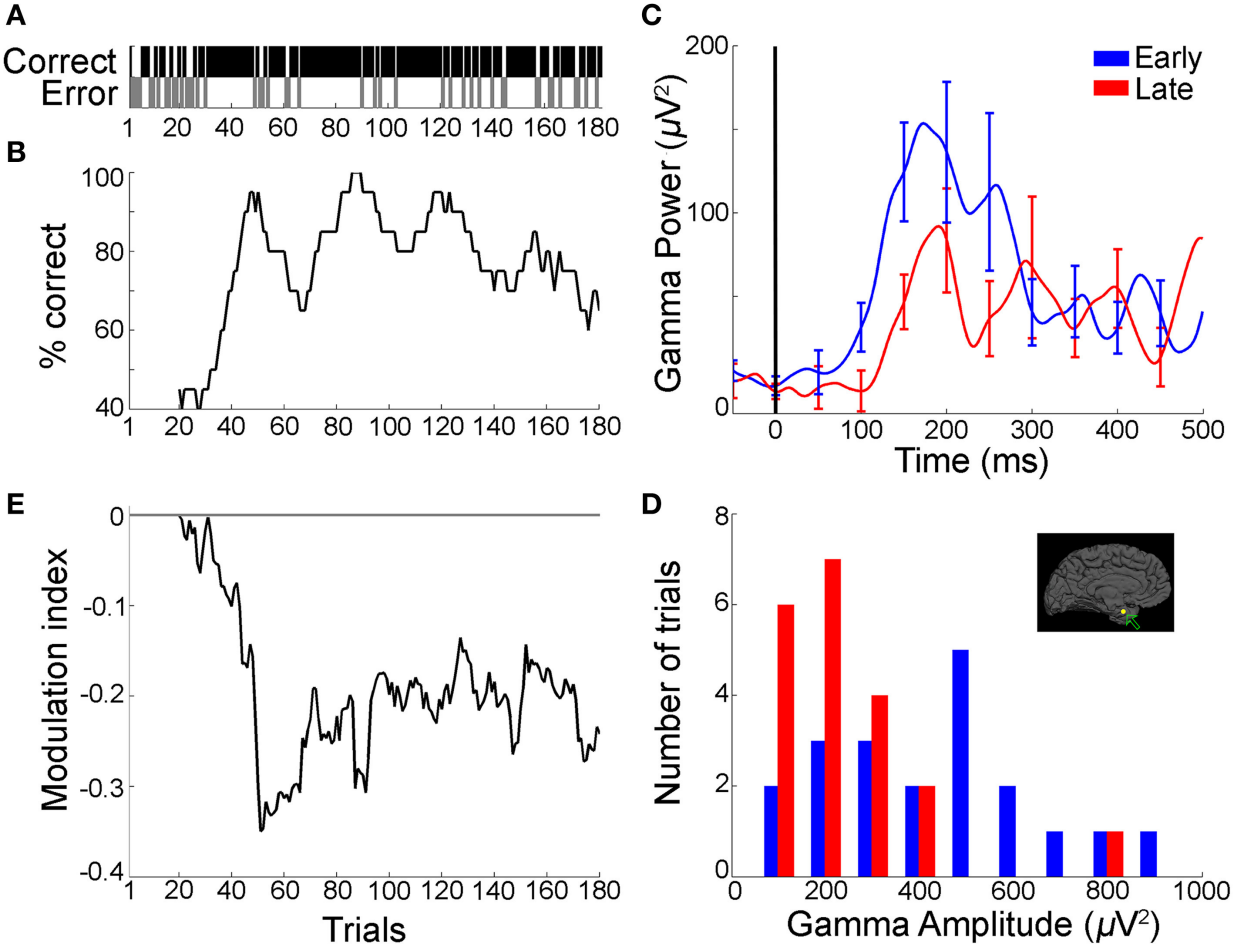
**Gamma frequency band power during the recall phase decreased with improved sequence learning over trials (example from Task 1)**. **(A)** Behavioral profile of a representative subject during sequence learning in Task 1 showing performance (correct, black; error, gray) at each trial (*n* = 182 trials). **(B)** Associated learning curve computed by using a sliding window of 20 trials stepped by one trial. **(C)** Mean gamma frequency band power (30–100 Hz) averaged over the first (“early,” blue) and last (“late,” red) 20 trials in the session, for an electrode in the left parahippocampal gyrus (Talairach coordinates: −19.2, −4.6, −30.2; inset in part d depicts electrode position). Data are aligned to choice screen presentation at *t* = 0. Error bars represent SEM and are shown every 50 ms for clarity. **(D)** Distribution of peak-to-peak gamma frequency band amplitudes (Gamma-band amplitude = *max* [Gamma-band power]—*min*[Gamma-band power] in the [0:500] ms window from choice screen onset) during early (blue) and late (red) trials. There was a significant reduction in gamma frequency band amplitudes in the late trials compared to the early trials (*p* = 0.003, rank sum test). There was no significant trend in the distribution of peak-to-peak broadband signals between early and late trials in the same electrode (*p* = 0.14, rank sum test). **(E)** Modulation index (MI) curve (difference between the mean gamma frequency band amplitudes in the early trials and subsequent trials, Materials and Methods), computed by using 20-trial sliding bins stepped by one trial, showing a decrease in gamma frequency band amplitude concomitant with the subject's performance improvement shown in *b* (mean MI: −0.19 ± 0.006 [mean ± SEM]). The horizontal line represents *MI* = 0 (i.e., no change in gamma frequency band amplitude).

Learning was evaluated as success in sequence order recall. *Task 1*: A trial was labeled as correct if the subject ordered all four images in the correct sequence. Out of 4! = 24 possible outcomes, only one was correct. *Task 2:* A trial was labeled as correct if the subject ordered the pairs of images presented on the choice screen in the correct sequence. The probability of making a correct choice for any given pair was 0.5. The learning curves for both tasks (Figures 1C,F, Supplementary Figure 1) were computed by calculating the percentage of correct choices in a sliding window of 10 trials shifted in steps of five trials. A session was classified as “learned” if the subject's performance reached ≥ 60% performance for at least two consecutive blocks of 10 trials. This threshold, referred to as “learning criterion” throughout the text, was chosen such that over the course of a session, the overall chance probability of correct responses in a 10 trial window was less than 0.01. Sessions that were below this learning criterion were classified as “not learned.” For Figures 2B, **4B** and Supplementary Figure 2b, the learning curves were computed using a sliding window of 20 trials shifted in steps of one trial.

In **Figure 5** (Task 2), the mean performance was computed for each individual sequence. The sequences were sorted by the mean behavioral performance. Trials corresponding to the two sequences with highest (lowest) mean percentage correct were referred to as the “high-performance” (“low-performance”) sequences. The results were similar when the sequences were sorted using the maximum percentage correct reached in a block of 10 trials instead of the mean. Reaction time (RT) for each trial was calculated as the time from the onset of the choice screen to the first button press.

### Spectral analyses

We analyzed IFP activity using two forms of spectral analysis (1) Morlet wavelets (2) Band pass filtering in conjunction with the Hilbert transform. Throughout the text, we only show results computed using the Morlet wavelets. In order to control for any bias due to the trade-off in frequency resolution in the wavelet transform, the analysis was repeated using the Hilbert transform. All analyses were done using custom-written code in MATLAB (Mathworks, Natick, MA). Let *_i_m*(*t*) represent the IFP at time *t* during trial *i* after notch filtering.

#### Morlet wavelets

We used Morlet wavelets (wave number of 6) to examine oscillatory activity across various frequency bands (1–100 Hz). This method provides a better compromise between time and frequency resolution than previously proposed methods using short-term Fourier transforms (Sinkkonen et al., 1995). Through the text, we focus on results from the wavelet analysis. The continuous wavelet transform (CWT) of *_i_m*(*t*) is the integral of the signal multiplied by scaled and shifted versions of a wavelet function and the mother wavelet ψ.

$$CWT(a,b)=\;\int_{-\infty }^{\infty }m(t)\frac{1}{\sqrt{a}}\psi^{*}\frac{\left(t-b\right)}{a}dt$$

where *a* and *b* are the scaling (width or frequency) and time localization of the shifting parameters, and (*) denotes the complex conjugate (Akay, 1998). ψ is the mother wavelet, which in this case is defined as: ψ(η) = π^−1/4^e^*i*ω_0η_^e^−η^2^/2^ (Morlet wavelet, where wavenumber ω_0_ = 6). The power envelope of the signal (*_i_p*_[*f*_1_, *f*_2_]_(*t*)) is computed as the squared absolute value of the wavelet transform |*CWT*(*a,b*)|^2^ between frequencies *f*_1_ and *f*_2_ (Akay, 1998).

#### Hilbert transform

The results were similar when using bandpass filtering and the Hilbert transform to analyze oscillatory patterns across these relatively broad frequency bands. We first filtered the raw signal in each frequency band using a second-order butterworth bandpass filter. Next, we applied the Hilbert transform, which yields a complex number (Freeman, 2004). We took the absolute value (analytic amplitude) of the Hilbert transform to extract the instantaneous power of the signal, *_i_p*_[*f*_1_,*f*_2_]_(*t*).

#### Task phases

We calculated the power across various frequency bands, across three main task phases: (1) Baseline phase ([0.500] ms before fixation onset) (2) Image presentation—[0.500] ms from image onset, four periods, one for each image in the sequence. The window encompassing all four image presentations is referred to as the Encoding phase. (3) Recall phase—[0.500] ms from the presentation of the choice screen. All analyses were based on the initial 500 ms after onset of the recall phase to capture the physiological responses correlated with recall before onset of the behavioral response (mean response time from the presentation of the choice screen to the first key press = 1.6 ± 0.6 s). All the task phases were 500 ms in duration for fair comparisons across epochs.

### Definitions for the variables shown in the figures

Let the average power between frequency *f*_1_ and *f*_2_ in trial *i* during the recall phase be

$${}_{i}p_{\left[f_{1},f_{2}\right]}\;(recall)=\frac{1}{500}\;\int_{-500}^{0}i\;p_{\left[f_{1},f_{2}\right]}\;(t)\;dt.$$

**Figure 3:**
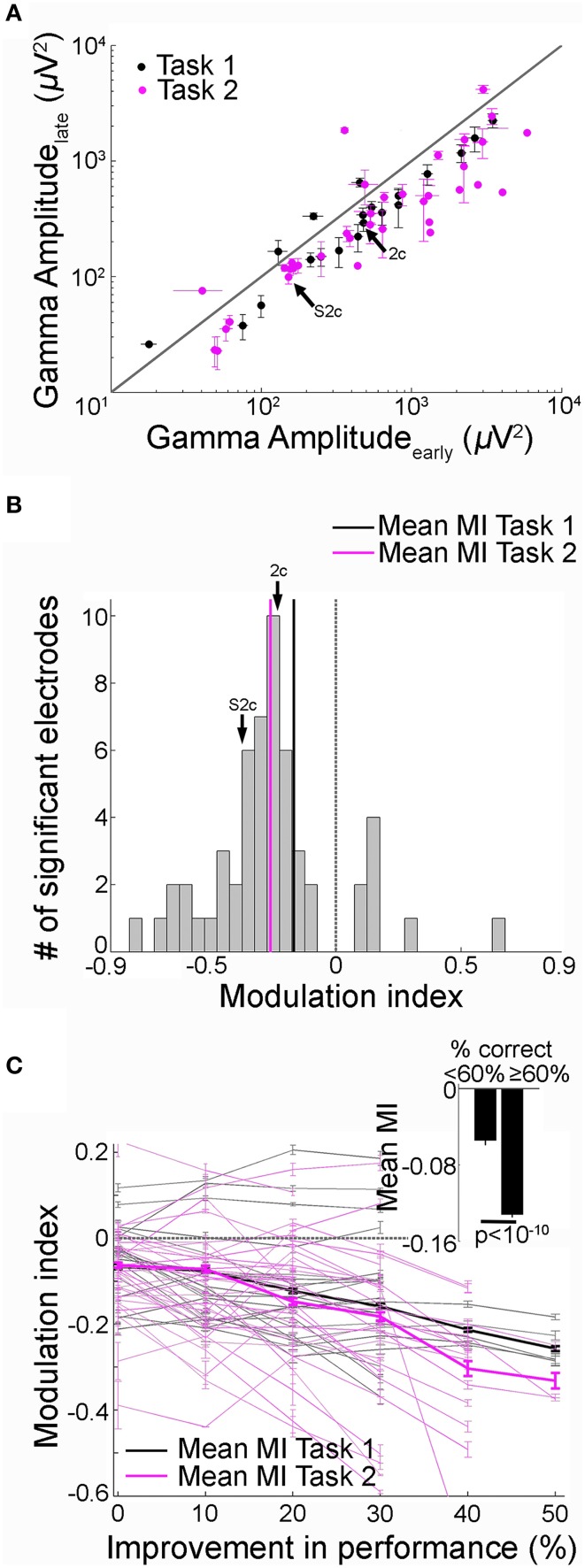
**Decrease in recall phase gamma frequency band amplitude over trials was directly correlated with learning**. **(A)** Gamma frequency band amplitudes in the late (y-axis) vs. early (x-axis) trials during the recall phase (black = Task 1; Pink = Task 2). Data from 51 electrodes that showed significant changes in gamma-band amplitudes with learning from 11 subjects that reached the behavioral learning criterion (Figure 1, Materials and Methods). The arrow points to the example electrode shown in Figure 2 and Supplementary Figure 2. Error bars represent SEM. The diagonal represents no change between early and late trials. **(B)** Distribution of modulation index (MI) values between early and late trials during the recall phase (both tasks combined). The *MI* values were predominantly negative (Task 1, 20 electrodes, −0.17 ± 0.04 [mean ± SEM], Task 2, 31 electrodes, −0.26 ± 0.02). The arrow points to the *MI* value for the example electrode in Figure 2 and Supplementary Figure 2. Dotted vertical line represents *MI* = 0. **(C)** MI as a function of behavioral performance improvement. Performance improvement along the x-axis was calculated as change in the percentage correct compared to the first block of 20 trials. Each point indicates the average MI for a given value of performance improvement. *MI* values and performance were computed by using a 20-trial sliding bin stepped by one trial. Each gray solid line represents an electrode from Task 1 and the thin pink line represents an electrode from Task 2; the thick lines show the average across electrodes. The dotted line indicates *MI* = 0. The Pearson correlation coefficient (*r*) between MI and behavioral performance improvement across 51 electrodes was −0.35 (*p* < 10^−5^). The range of *MI* values were restricted to (−0.6 0.2) for visualization. *MI* values outside this range from 2/51 electrodes are not shown. Mean *MI* values for low (<60%) and high (≥ 60%) performance 20-trial bins are shown in the inset (*p* < 10^−10^, *t*-test). Error bars denote SEM.

**Figure 4:**
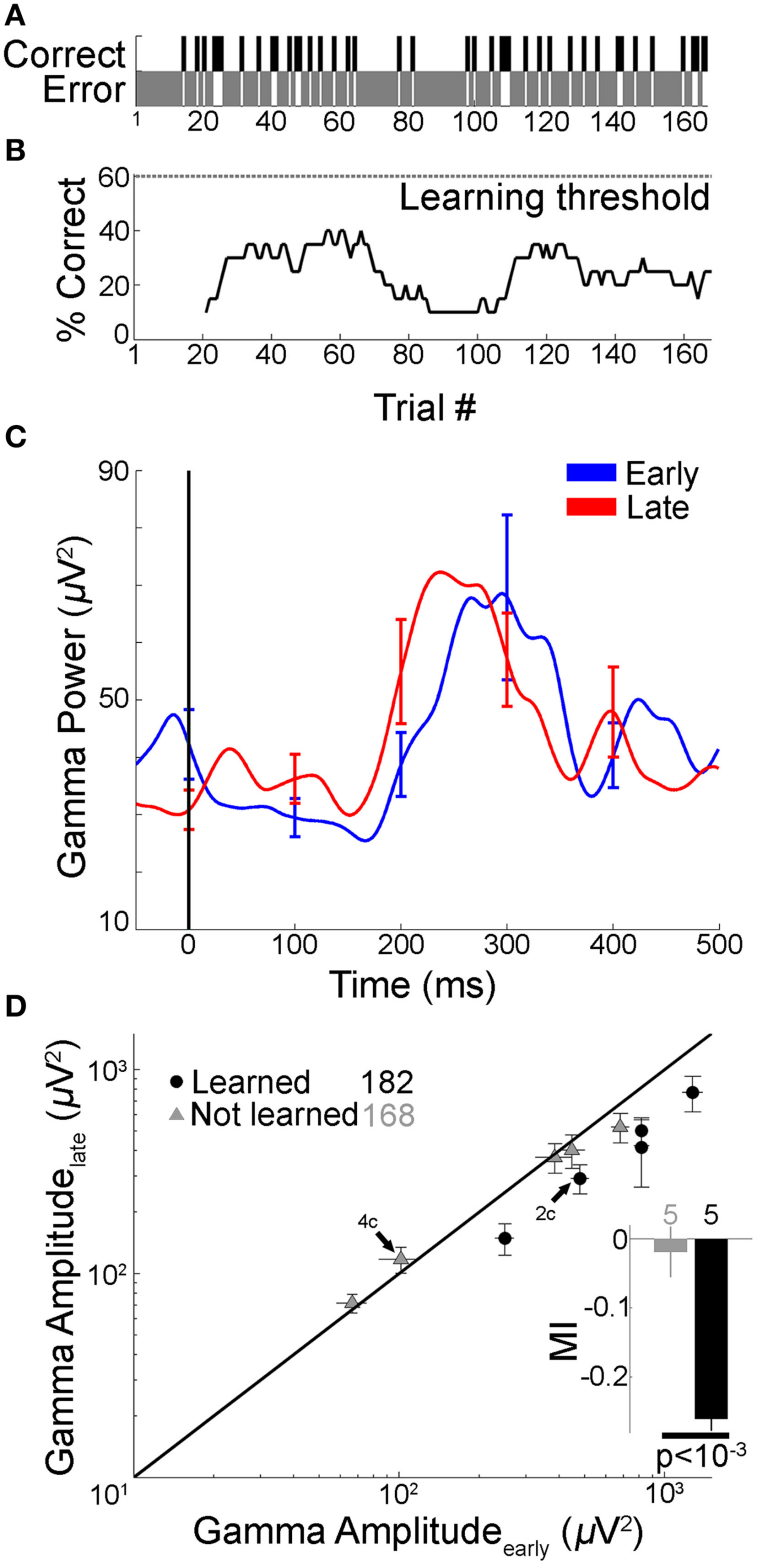
**Gamma frequency band amplitude remained unchanged if subject did not learn**. In four subjects that did not learn the tasks, <1% of the electrodes showed changes in gamma band amplitude with learning. In order to directly compare changes in gamma-band amplitude with behavioral performance in the same electrodes we report results from one example subject that participated in two sessions of Task 1: in one of those sessions the learning criterion was not reached. **(A,B)** Performance for a subject who participated in two sessions of Task 1 (green lines in Figure 1C). In one session, the subject reached the learning criterion (maximum performance was >90%, shown in Figures 2A,B). In the other session, shown here, the subject did not reach the learning criterion (maximum performance <40%). The format is the same as in Figures 2A,B. **(C)** Mean gamma frequency band power averaged over the early and late trial blocks (each 20 trials), for the same electrode in Figure 2C, shown here during the session where the learning criterion was not reached. Figure format is the same as Figure 2C. The gamma frequency band amplitude was not significantly different between early and late trials (*p* = 0.32, rank sum test). **(D)** Five out of 69 electrodes in this subject showed significant changes in gamma frequency band amplitudes (*p* < 0.01) between early and late trials in the session where there was learning compared to zero electrodes in the session where there was no learning. Here we compare the gamma frequency band amplitudes during the recall phase for the same five electrodes between the “not learned” session (gray triangles, 168 trials) and the “learned” session (black circles, 182 trials) in the late (y-axis) vs. early trials (x-axis). Arrows point to the example electrode in Figures 2C, 4C. Inset shows the average MI in “not learned” and “learned” blocks (*p* < 10^−3^, rank sum test).

Figures 2C, **4C** and Supplementary Figure 2c show 〈*p*_[30,100]_(*t*)〉_*early*_ and 〈*p*_[30, 100]_(*t*)〉_*late*_ where <> denotes averaging across trials and <>_early/late_ indicates average over the first/last 20 trials in a session. To summarize the dynamic responses in a single number, we defined the amplitude in a time interval *I* as the peak-to-peak power:

$${}_{i}A_{\left[f_{1},f_{2}\right]}[I]=\underset{I}{\max}\;\left({}_{i}\;p_{\left[f_{1},f_{2}\right]}\left(t\right)\right)-\underset{I}{\min}\;\left({}_{i}\;p_{\left[f_{1},f_{2}\right]}\left(t\right)\right)$$

with *t* ∈ *I*. Figure 2D and Supplementary Figure 2d show the distribution of *_i_A*_[30, 100]_[*recall*] for early and late trials. Figures 3A, 4D show 〈*A*_[30, 100]_[*recall*]〉_*late*_ vs. 〈*A*_[30, 100]_[*recall*]〉_*early*_ for multiple electrodes. To measure the relation between power amplitudes and learning, a modulation index (MI) was defined as:

$$MI_{\left[f_{1},f_{2}\right]}\left(block,I\right)=\frac{{\langle A_{\left[f_{1},f_{2}\right]}\left(I\right)\rangle }_{block}\;-\;{\langle A_{\left[f_{1},f_{2}\right]}\left(I\right)\rangle }_{early}}{{\langle A_{\left[f_{1},f_{2}\right]}\left(I\right)\rangle }_{block}\;+\;{\langle A_{\left[f_{1},f_{2}\right]}\left(I\right)\rangle }_{early}}$$

where block is a set of 20 trials. An *MI* value of zero implies that the amplitude in a block of trials is the same as that during the first block of 20 trials; that is, *MI* = 0 implies that there is no change in amplitude with learning. Figure 3B shows *MI*_[30,100]_(*late*, *recall*) for all trials in the session. Figure 2E and Supplementary Figure 2e show the MI curve obtained by computing MI in blocks of 20 trials with a sliding window of 1. Figure 3C shows 〈*MI*_[30,100]_(*block*, *recall*)〉, where <> denotes average across trials with a given performance improvement for each electrode (thin lines in Figure 3C). Performance improvement was calculated as the change in the percentage of correct trials for a given set of trials compared to the early trials. Figure 3C and Supplementary Figure 5b show 〈*MI*_[30,100]_(*block*, *recall*)〉 where <> denotes the average across electrodes. Supplementary Figure 4 shows 〈*MI*_[*f*1,*f*2]_(*block*, *recall*)〉 where <> denotes the average across electrodes and frequencies [*f*1 *f*2] were (a) [1 30], (b) [10 30], (c) [30 50], (d) [70–100] Hz respectively. Thick solid lines in Figure 3C show the average across all individual curves (individual electrodes) from each task. Linear correlations were measured using the Pearson correlation coefficient.

**Figure 5:**
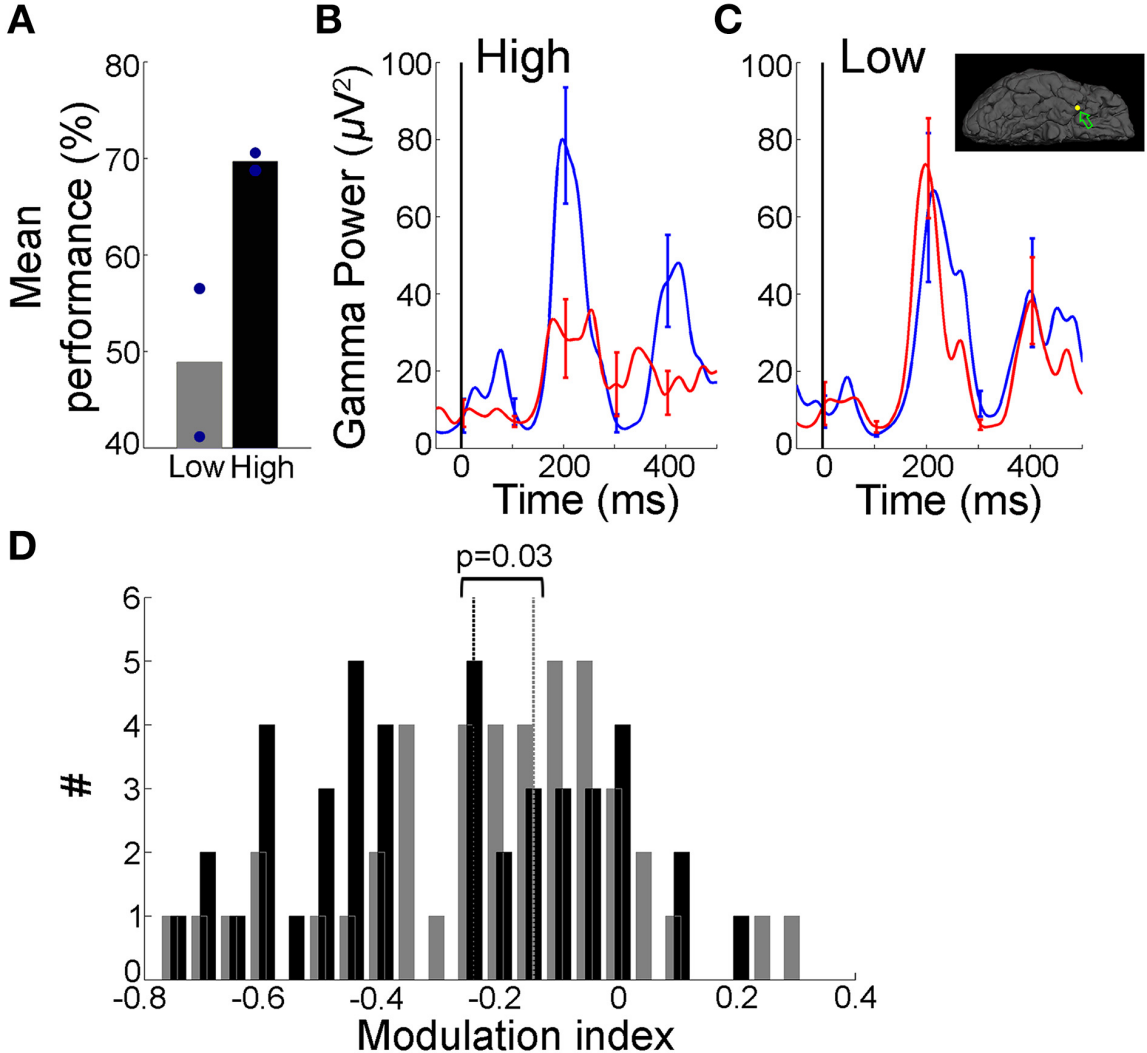
**Decrease in gamma band activity was restricted to learned sequences**. **(A)** Performance across different sequences within a single level for an example subject that participated in Task 2. This subject learned five, eight-image long sequences during the course of this level. Behavioral performance for each sequence was calculated separately and sequences were ranked by performance (“low”/”high” = two sequences with lowest/highest mean percentage correct; circles indicate individual sequences). **(B,C)** Mean gamma frequency band power averaged over the early (blue) and late (red) trials (each 15 trials), for “High” sequences **(B)** and “Low” sequences **(C)** for an electrode in the right temporal pole (Talairach coordinates: 28.6, 3.7, −37.0; inset in **(C)** depicts electrode position). Figure format is the same as Figure 2C. The gamma frequency band amplitude was significantly different between early and late trials in **(C)** (*p* = 0.02, rank sum test, *MI* = −0.11) but not in **(C)** (*p* = 0.38, rank sum test, *MI* = 0.003). **(D)** Distribution of Modulation index values for sequences showing “High” (black) and “Low” (gray) behavioral performance across four subjects that successfully learned Task 2 and with ≥ 30 trials in the “High”/“Low” categories (dotted lines indicate median values). There was a significant difference in the *MI* values (*p* = 0.03, rank sum test). The mean performance was 88 ± 10% for “High” and 58 ± 16% for “Low” sequences. Data from 22/31 electrodes that showed significant differences between early and late trials in Task 2.

Analyses at the level of individual sequences are shown in Figure 5 (Task 2). Figures 5B,C show 〈*p*_[30,100]_(*t*)〉_*early*_ and 〈*p*_[30,100]_ (*t*)〉_*late*_ where <>_early/late_ indicates average over the first/last 15 trials in high-performance and low-performance sequences. Figure 5D shows *MI*_[30,100]_(*late*, *recall*) for each individual sequence in a level where late indicates the last 15 trials of the sequence. For pooling data across all subjects that learned Task 2 in Figure 5D, only levels with ≥ 30 trials in the high and low performance categories were included in the analysis.

**Figure 6:**
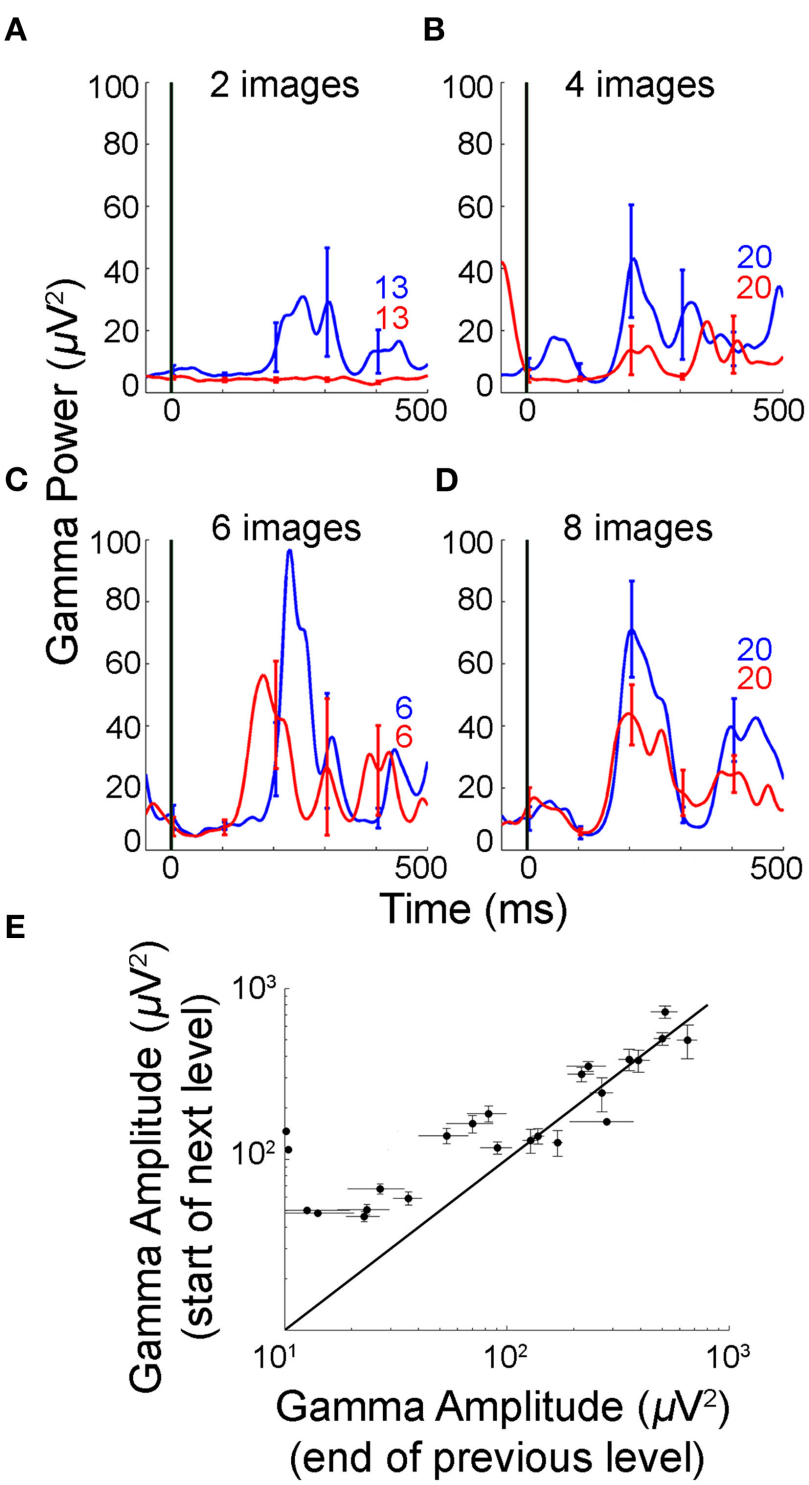
**Gamma frequency band amplitude was reset at the start of each level in Task 2**. **(A–D)** Mean gamma frequency band power averaged over early and late trials in multiple levels for the same electrode in Figures 5B,C (numbers of trials shown in each subplot). The subject participated in a single experimental session consisting of four levels of increasing sequence length. The interval between levels ranged from 8 to 183 s. The maximum performance in any bin of 10 trials was 100% in level 1 (27 trials), 100% in level 2 (96 trials), 80% in level 3 (13 trials), and 70% in level 4 (80 trials). The mean gamma frequency band amplitude decreased significantly between “early” and “late” trials within levels 1, 2, and 4 as the sequences were learned (*p* < 0.01, rank sum test). Level 3 had a small number of trials since the subject learned the sequences rapidly and significance was not reached even though the trend is the same as in the other levels. The mean gamma frequency band amplitude did not continue to decrease across levels but instead was reset to a new and higher value. **(E)** Gamma frequency band amplitudes in the last 20 trials for the previous level (x-axis) vs. first 20 trials in the next level (y-axis) during the recall phase This figure includes *N* = 3 out of four subjects that reached the learning criterion in Task 2 and participated in at least two levels with ≥ 20 trials. The diagonal represents no change in gamma frequency band amplitudes between the last 20 trials of the preceding level and the first 20 trials in the next level. Error bars represent SEM.

Gamma frequency band analysis was performed separately for each level in Task 2. Figures 6A–D show 〈*p*_[30,100]_(*t*)〉_*early*_ and 〈*p*_[30,100]_(*t*)〉_*late*_ where <>_early/late_ indicates averaging over the first/last trials restricted to each level. Figure 6E shows 〈*A*_[30,100]_[*recall*]〉_*end*_ vs. 〈*A*_[30,100]_[*recall*]〉_*start*_ for multiple electrodes where <>_end/start_ indicates average over the last/first 20 trials in a level.

### Statistical analyses

For each electrode, gamma-band amplitudes for each trial were calculated and to compare changes in gamma-band amplitudes with learning over trials, the distribution of gamma-band amplitudes in the early (first 20 trials) and late (last 20 trials) trials in a session were compared using the non-parametric rank sum test (*p* < 0.01 was considered significant, i.e., learning-modulated electrodes). Since Task 2 had two recall phases for levels 2–4, distribution of gamma-band amplitudes for each electrode in the early and late trials were compared during both “recall” phases. Learning-modulated electrodes showed significant changes in gamma in at least one of the “recall” phases (*p* < 0.01). We repeated the analysis described above for the gamma-band, for four other frequency bands [1–10], [10–30], [30–50], and [70–100] Hz (Supplementary Figure 4). In Figure 3C, we compared the distribution of correlation coefficients between *MI* and performance improvement obtained from 51 electrodes against a null distribution obtained from 500 shuffles of trial order. These surrogate data was analyzed using the same methods and criteria applied to the real data. To control for the bias in the numbers of error trials between early and late trials, the analysis was repeated using only the correct trials within a session. The same statistical criteria were used to determine significance.

## Results

We analyzed electrophysiological field potential activity from 1142 intracranial electrodes implanted in 14 epilepsy subjects while they performed one of two temporal-order recall tasks. Both tasks involved remembering and reporting the temporal order of sequentially presented images (Figure 1, Materials and Methods). We recorded IFPs while subjects performed both tasks (see Table 1 for electrode locations). Learning was defined as the improvement in sequence recall performance over trials. The frequency spectrum of the IFP on each trial was calculated and we asked whether the modulation in power in the gamma frequency band (30–100 Hz) during sequence recall was correlated with learning.

### Tasks and behavioral performance

In Task 1, subjects were presented with a sequence of four images, (referred to as the Encoding phase, Figure 1A). After a short delay, subjects were presented with a choice screen displaying all four images on the screen at once (referred to as the Recall phase). Subjects had to report the order in which the images had been presented using four buttons on a gamepad. Subjects were presented with multiple overlapping sequences in pseudorandom order (Figure 1B) and each subject participated in 1 or 2 sessions with different images (Materials and Methods). Performance in a trial was labeled *correct* if the subject was able to report the correct order for *all* four images in a sequence. Subjects learned the order of multiple sequences through repeated recall. Throughout the manuscript, we examine the relationship between neural signals and this behavioral improvement in recall performance via repetition. Successful learning was defined as reaching a recall performance of ≥ 60% correct for at least two consecutive blocks of 10 trials. This threshold is referred to as the learning criterion and was chosen such that the overall probability of correct responses in a 10 trial window during the task by chance was less than 0.01 (Materials and Methods). Seven (of eight) subjects were able to successfully reach the learning criterion (Figure 1C). One subject participated in two sessions and was successfully able to reach learning criterion in one session. The session in which this subject did not learn the task is discussed separately in Figure 4. On average, subjects needed 28 ± 11 [mean ± standard deviation (SD)] trials to reach the learning criterion (Supplementary Figure 1). Performance for recall of the first image in the sequence was higher than for the last image or intermediate combinations (Supplementary Figure 1a), consistent with the notion that the first events in a sequence are remembered more accurately (Tulving, 2002). Subjects that did not reach learning criterion were equally likely to fail in any of the three consecutive image pairs in the sequence (Supplementary Figure 1a). The behavioral learning rate was comparable in subjects with epilepsy (Supplementary Figure 1a) vs. healthy volunteers (Supplementary Figure 1b). On average, healthy volunteers needed 27 ± 10 (mean ± SD) trials to reach the learning criterion.

In addition to learning about the object sequence, it is conceivable that subjects learnt about the contingencies of the task over time and improved their motor responses, which required using four different buttons. To examine this possibility, we compared performance improvement and the RTs. If the improvement in sequence recall performance were driven purely by improved motor skills, we would expect a strong correlation between recall performance and RTs. However, RTs were not correlated with recall performance (*r* = −0.06). We also conducted a separate task where we simplified the motor response to a binary decision, as described next.

We considered a variant of the task (Task 2) with three main differences (Figures 1D,E; Materials and Methods): (i) sequences were *not* overlapping (to examine learning for different individual sequences); (ii) there were separate levels consisting of different sequence lengths from 2 to 8 images (to examine learning at different difficulty levels), and (iii) order recall was evaluated via binary comparisons (to reduce the motor complexity of the task). In contrast to Task 1, subjects were presented with multiple non-overlapping sequences in pseudorandom order within each level (Figure 1E). Performance in a trial was labeled *correct* if the subject was able to report the correct order for *all* presented pairs of images in the recall phase. Four (of 6) subjects were able to successfully reach the learning criterion (Figure 1F). On average, subjects needed 25 ± 22 (mean ± SD) trials to reach the learning criterion (Supplementary Figure 1c). On average, healthy volunteers needed 20 ± 20 (mean ± SD) trials to reach the learning criterion in Task 2 (Supplementary Figure 1d). Recall performance was not correlated with the RTs in Task 2 (*r* = 0.18).

### Gamma frequency band amplitude during the recall phase decreased over trials with improved sequence recall performance

To evaluate the physiological correlates of sequence recall over trials, we compared the gamma frequency band power with the behavioral improvement in percentage correct recall performance during the task. It is important to note that the gamma band activity analyzed in this study are broadband signals, which span the gamma range but do not have a distinct peak (Miller et al., 2009; Ray and Maunsell, 2011), and should be distinguished from the narrowband gamma oscillations, which are indicated by a peak in the spectral response (Lopes da Silva, 2013). The improvement in recall performance over the course of a session in Task 1 for an example subject is shown in Figures 2A,B. Recall improved as the subject viewed the sequences repeatedly, with the subject reaching >80% correct performance from an initial performance of 40% in the first 20 trials. We investigated whether these behavioral changes were manifested in the gamma frequency band power during the recall phase ([0.500 ms] from the choice screen onset, Materials and Methods). Figure 2C depicts an example electrode in the left parahippocampal gyrus (Talairach coordinates: -19.2, -4.6, -30.2) that showed a decrease in power in the gamma frequency band during the recall phase when comparing the first 20 trials (“early” trials, blue) vs. the last 20 trials (“late” trials, red) within a session. We observed a decrease in gamma frequency band power from early to late trials, particularly during the 100–300 ms after onset of the recall phase (Figure 2C). To summarize the physiological responses, we defined the “gamma-band amplitude” as the peak-to-peak value of gamma frequency band power during the recall phase (max[*Gamma-band power*]—min[*Gamma-band power*], Materials and Methods). The distribution of gamma frequency band amplitudes in the recall phase showed a shift toward lower values during late trials compared to early trials (Figure 2D, *p* = 0.003, rank sum test). To directly compare the changes in gamma frequency band amplitude and performance improvement, we calculated a modulation index (MI), defined as the difference between the mean gamma frequency band amplitude in a block of 20 trials and the corresponding value in the first 20 trials (Materials and Methods). Positive (negative) MI values indicate increase (decrease) in gamma frequency band amplitude with respect to the early trials. The changes in the modulation index over the course of multiple trials (Figure 2E) paralleled the concomitant performance changes observed at the behavioral level (Figure 2B). In particular, large changes in performance seemed to be accompanied by large changes in the modulation index (around trial number 40 in Figures 2B,E). These changes in gamma-band amplitude could not be attributed to broadband voltage fluctuations in the signal over time (the peak-to-peak broadband IFP voltage in the recall phase remained unchanged over trials, *p* = 0.14, rank sum test). Supplementary Figure 2 depicts an example electrode in the temporal pole recorded during Task 2, showing a similar trend of decreased gamma-band amplitudes in the recall phase with improved performance.

The gamma band power increased in the recall phase (Figure 2C, Supplementary Figures 2c, 3), but the degree of enhancement of power in the gamma frequency band decreased over trials with learning (Figures 2C–E, Supplementary Figures 2c–e). This reduction in gamma power enhancement over trials is represented as a decrease in gamma amplitudes.

We recorded activity from 917 electrodes in the 11 subjects that showed successful learning over trials in either Task 1 or 2. We identified 51 electrodes (5.5% of the total, 20 in Task 1, 31 in Task 2) that showed significant changes in gamma frequency band amplitude in the recall phase between early and late trials (*p* < 0.01 rank sum test, Materials and Methods). As illustrated in the example electrodes in Figure 2, Supplementary Figure 2, 43/51 (84%) of these electrodes showed a decrease in gamma-band amplitude during the recall phase in the late trials compared to the early trials both in Task 1 and Task 2 (Figure 3A). The mean modulation index (MI) for these electrodes was −0.22 ± 0.03 (Figure 3B, mean ± standard error of mean (SEM); Task 1: −0.17 ± 0.04; Task 2: −0.26 ± 0.02).

The number of correct trials was larger in late compared to early trials (as discussed under Task Performance above when showing behavioral improvement over trials). We asked whether the changes in the gamma band neural signals could be a result of the larger proportion of error trials or whether those changes would still be evident when comparing only correct trials. We repeated the analyses using only the first 20 and the last 20 correct trials within a session. Thirty-five electrodes (3.8% of the total) showed significant differences in gamma amplitudes in the recall phase between early and late correct trials; most (85%) of these electrodes showed a decrease in gamma-band amplitude (mean *MI* = −0.17 ± 0.03).

Departures in MI from 0 during the recall phase were expected given that these electrodes were selected based on the gamma-band amplitude changes during this phase. To evaluate the whether these results were specific to the recall phase or could be attributed to global changes over trials, we repeated the analyses during the encoding and baseline phases. In contrast to the observations during the recall phase of the task, no significant changes were observed in the gamma-band amplitude computed during the encoding (mean *MI* = −0.07 ± 0.01) or baseline (mean *MI* = −0.04 ± 0.03) phases of the task.

To directly relate gamma-band amplitude changes to recall performance or learning over trials, we plotted the *MI* values as a function of the behavioral recall performance improvement calculated as the difference in percentage correct between early trials and subsequent 20-trial bins (Figure 3C, Materials and Methods). The negative correlation in Figure 3C reflected the gradual decrease in gamma frequency band power in the recall phase with improved recall behavior (*r* = −0.35, *p* < 10^−5^). We evaluated the statistical significance of the correlation coefficients *r* between MI and performance improvement by comparing them against the distribution of *r* values calculated from 500 iterations where trial order was randomly shuffled (Materials and Methods). We found that the correlation coefficient values obtained here could not have been derived from the shuffled null distribution (*p* < 0.01). Further, the mean *MI* values in 20-trial bins with low recall performance (<60%) were significantly different from trial bins with high recall performance (≥ 60%), further supporting the finding that gamma frequency band amplitudes decrease with learning (inset Figure 3C, *p* < 10^−10^, *t*-test). Results were similar when gamma frequency band power was re-calculated using the absolute value of the Hilbert transform (Materials and Methods). To investigate whether the relationship between power amplitude and recall performance was limited to the gamma-band, we repeated the analyses for four other frequency bands (Supplementary Figure 4). Frequency bands below 30 Hz did not show any consistent relationship between MI and improvement in recall performance (Supplementary Figures 4a,b). Frequency bands [30–50] Hz and [70–100] Hz showed significant correlation between MI and performance improvement (Supplementary Figures 4c,d). Hence, in all subsequent analyses, we have used the combined [30–100] Hz band as the gamma frequency band.

The placement of the electrodes was exclusively based on clinical criteria (Materials and Methods), leading to the possibility of examining multiple different locations. We co-registered the preoperative MRI with the postoperative CT images and mapped each electrode onto one of 74 brain locations (Destrieux et al., 2010) (Tables 1, 2). As expected given the sampling procedure, most of the electrodes did not show activity changes during the task (Table 1). For each area where we had at least 10 electrodes, we asked whether the number of electrodes showing differential gamma power between early and late trials was different from that expected number under the null hypothesis given the sampling distribution. The parahippocampal gyrus and the middle temporal gyrus showed an enhanced proportion of electrodes with learning-related modulation of gamma frequency band amplitude during the recall phase (Supplementary Figure 5).

**Table 2:** **Location and statistics of 51 electrodes that showed significant (*p* < 0.01, rank sum test) changes in gamma band amplitudes with learning (*N* = 11 subjects that learned Task 1 and 2)**.

**Region**	**Talairach**			***P*-value**
Parahippocampal gyrus	−27.8	−8.7	−39.0	0.0043
Parahippocampal gyrus	30.5	8.8	−36.8	0.0028
Parahippocampal gyrus	−19.1	−3.3	−24.4	0.01
Parahippocampal gyrus (Figure 2)	−19.2	−4.6	−30.2	0.003
Parahippocampal gyrus	−28.0	−5.2	−38.6	0.0043
Parahippocampal gyrus	−23.5	−8.0	−34.0	0.0011
Parahippocampal gyrus	−29.0	−14.9	−35.2	0.0028
Parahippocampal gyrus	20.8	−21.7	−22.9	0.0006
Middle temporal gyrus	−61.1	−15.6	−24.1	0.0033
Middle temporal gyrus	−48.2	4.2	−34.0	0.0031
Middle temporal gyrus	−67.0	−12.9	−11.9	0.002
Middle temporal gyrus	−47.6	−4.5	−36.5	0.008
Middle temporal gyrus	−53.1	−8.4	−33.6	0.004
Middle temporal gyrus	−56.7	−1.0	−26.6	0.007
Middle temporal gyrus	−51.5	4.8	−17.7	0.008
Middle temporal gyrus	−58.6	−16.6	−26.4	0.0021
Temporal pole	−40.6	−8.4	−38.2	0.005
Temporal pole	−44.6	16.9	−18.3	0.00003
Temporal pole	−39.7	13.9	−27.7	0.007
Temporal pole (Figure S2, Figures 5B,C, 6A-D)	28.6	3.7	−37.0	0.009
Temporal pole	30.8	8.6	−34.4	0.0066
Temporal pole	28.6	3.7	−37.0	0.007
Temporal pole	32.6	−4.1	−41.2	0.0018
Orbital gyrus	21.5	49.2	−27.0	0.006
Orbital gyrus	32.2	63.5	−20.1	0.0098
Orbital gyrus	8.4	27.4	−26.7	0.0003
Orbital gyrus	14.2	53.2	−25.7	0.0006
Inferior temporal gyrus	45.9	21.1	48.7	0.0047
Inferior temporal gyrus	−47.6	−16.9	−33.8	0.0016
Inferior temporal gyrus	−50.3	−52.1	−21.3	0.0028
Middle frontal gyrus	38.8	46.9	16.6	0.0004
Middle frontal gyrus	51.2	40.8	9.8	0.004
Middle frontal gyrus	35.7	63.7	−11.9	0.0084
Precuneus	1.1	−80.9	19.4	0.0077
Precuneus	2.8	−89.6	11.3	0.009
Precuneus	0.3	−75.2	25.3	0.0051
Triangular part of the inferior frontal gyrus	−45.5	−1.6	54.6	0.0043
Triangular part of the inferior frontal gyrus	−55.4	36.2	−3.9	0.0066
Triangular part of the inferior frontal gyrus	57.4	20.1	3.4	0.0066
Superior temporal gyrus	−44.8	16.9	−22.5	0.006
Superior temporal gyrus	−59.9	0.7	−11.4	0.0026
Superior temporal sulcus	55.2	−62.2	2.7	0.0051
Middle occipital gyrus	−19.8	−92.3	18.7	0.0006
Middle occipital gyrus	−40.0	−79.0	22.9	0.005
Postcentral gyrus	−42.4	−16.0	58.6	0.0047
Postcentral gyrus	60.1	−11.5	32.2	0.0098
Frontomarginal gyrus (of Wernicke) and sulcus	20.3	59.5	−14.9	0.009
Precentral gyrus	24.6	−41.5	64.6	0.0066
Subcentral gyrus (central operculum) and sulci	−65.4	−12.8	20.9	0.0015
Occipital pole	16.0	−97.2	1.8	0.0013
No accurate mapping onto the brain surface				0.005

### Gamma frequency band amplitude did *not* change over trials in the absence of learning

Changes in gamma frequency over trials could be the result of non-specific effects such as attentional decline or subject fatigue during the course of the experiment. To the extent that attentional decline or similar effects would manifest themselves in all phases of the task, the lack of changes in the gamma frequency band during the encoding and baseline phases described above argue against a non-specific interpretation. To further evaluate the relationship between gamma frequency band amplitude changes during the recall phase and learning, we investigated those few sessions where subjects did not reach the learning criterion of 60% performance (2 subjects in Task 1 and 2 subjects in Task 2, lines marked by arrows in Figures 1C,F). Only three of the 306 electrodes (<1%) in these 4 subjects showed significant changes in gamma frequency band amplitude over trials during the recall phase. Given the distribution of number of electrodes across subjects, we asked whether we could expect this small fraction of electrodes that showed significant changes in gamma frequency band amplitude over trials in subjects that did not learn the task, by chance. We computed the fraction of electrodes that showed a change in the gamma frequency band over trials (Materials and Methods) in those subjects that successfully learned, *f_L_*, and the corresponding fraction in those subjects that did not learn the task, *f_NL_*. Our null hypothesis was that these two fractions were the same. We tested this null hypothesis by comparing *f_L_*-*f_NL_* against the distribution obtained by randomizing the label indicating whether a subject learned the task or not. The actual difference (*f_L_*-*f_NL_*) was significantly different from the null distribution (*p* < 0.05).

Across-subject comparisons are difficult to interpret given that the electrode locations differ across patients. Given the small numbers of significant electrodes in subjects that did not learn the tasks, in order to directly compare changes in gamma-band amplitudes with learning performance in the same electrodes we report results from one subject that participated in two sessions of Task 1: in one of those sessions the learning criterion was not reached (Figures 4A,B, “not learned”) while in the other session the learning criterion was reached (shown in Figures 2A,B, “learned”). The activity during the “not learned” session for the same subject and the same example electrode illustrated in Figure 2C is shown in Figure 4C. In the absence of learning, gamma frequency band power in the recall phase remained unchanged between “early” and “late” trials (Figure 4C, *p* = 0.32, rank sum test). In this subject, there were five electrodes that showed significant changes in gamma frequency band amplitude in the “learned” session. None of electrodes showed significant changes in gamma frequency band amplitude in the “not learned” session. To directly compare the physiological responses in the same subject and electrodes in the “learned” vs. “not learned” session, we plotted the gamma frequency band amplitude for these five electrodes in the late vs. early trials (Figure 4D). Whereas the gamma frequency band amplitude was consistently lower in the late trials compared to early trials during the learned session, no such relationship was observed in the absence of learning. The average MI for the same 5 electrodes during the “not learned session” was 0.01 ± 0.03 (mean ± SEM) compared to −0.26 ± 0.01 in the “learned session” (Figure 4D inset).

### Decrease in gamma band amplitude over trials was most pronounced for sequences showing higher behavioral performance

The analysis in Figures 2–4 does not distinguish between individual sequences but considers overall learning of multiple sequences within a session. To further examine the relationship between gamma band amplitude changes and learning, we examined performance and physiological responses for individual sequences within each level in Task 2. Each subject learned multiple non-overlapping sequences within a level. Because different sequences were randomly interleaved, it is difficult to ascribe changes in performance or physiological responses across sequences to non-specific global factors. We considered the two sequences with the highest percentage correct (“high-performance sequences”) and the two sequences with the lowest percentage correct (“low-performance sequences”) within each level (e.g., Figure 5A). This analysis was not possible for Task 1 because of the sequence overlap. It should be emphasized that this separation between “high” and “low” performance sequences is purely based on the behavioral performance and not on the physiological responses. Figures 5B,C depicts the gamma frequency band power in early and late trials for high-performance and low-performance sequences for an example electrode in the right temporal pole (Talairach coordinates: 28.6, 3.7, -37.0). There was a significant decrease in gamma frequency band power between “early” and “late” trials in the high-performance sequences (*p* = 0.02, Figure 5B) but not in the low-performance sequences (*p* = 0.38, Figure 5C). Pooling data from 4 subjects that learned Task 2, the *MI* values for high-performance sequences were significantly lower than those for low-performance sequences (Figure 5D, *p* = 0.03, rank sum test). These changes could not be attributed to faster response times: RTs were comparable for low and high performance sequences (*p* = 0.37, rank sum test). Further, the *MI* values for high-performance sequences were significantly more negative than the low-performance sequences when the analysis was repeated using only the correct trials within a sequence (*p* = 0.03, rank sum test), hence ruling out the possibility of the decrease in gamma amplitudes being explained by the different numbers of correct trials.

### Reset in gamma band power on presentation of novel sequences

In Task 2, subjects moved to more complex levels (i.e., longer sequences) upon mastering sequences of a given length (Materials and Methods). We compared gamma activity when subjects transitioned from learnt sequences at one level to novel sequences at the next level. Figures 6A–D shows the gamma frequency band power for early and late trials for the same electrode shown in Figures 6B,C during levels 1 through 4 (2–8 images). Consistent with the results described in Figures 2, 3 and Supplementary Figure 2, gamma frequency band amplitude significantly decreased as the subject's performance improved over trials within each level. Gamma frequency power did not decrease monotonically from the beginning of the experiment in level 1 to the end of the experiment in level 4. Interestingly, after the late trials in a given level, the gamma band amplitudes increased upon beginning of another level with new sequences (e.g., compare red trace in Figure 6A vs. blue trace in Figure 6B). Three subjects that participated in Task 2 (out of 4 subjects that reached the learning criterion in Task 2) performed at least two levels of the task with ≥ 20 trials in each level. We compared the average gamma frequency band amplitude in the first 20 trials of a given level (“Next”) vs. the last 20 trials of the preceding level (“Previous”) for 24 electrodes in these 3 subjects (Figure 6E). The gamma frequency band amplitudes for the first trials in the “Next” level were significantly higher than the ones in the last trials of the “Previous” level (*p* < 0.001, one-tailed rank sum test). These observations show that that gamma frequency band amplitude did not decrease monotonically during the entire experimental session when the sequences and levels changed, but instead was reset to a different and higher value at the start of each level. The observed gamma amplitude reset could be an indicator of novelty (Xiang and Brown, 1998) or higher cognitive load (more complex levels, Howard et al., 2003).

## Discussion

Subjects performed a sequence recall task by arranging serially presented items in the correct temporal order. We computed a learning curve for each subject by defining the probability of a correct choice as a function of trial number and compared this psychometric curve to physiological changes evaluated from IFP recordings filtered in the gamma frequency band during the recall phase. Subjects improved their recall performance with repetition (Figures 1C,F, Supplementary Figure 1). Concomitant with these behavioral changes, we observed a decrease in the amplitude of IFPs in the gamma frequency band during the recall phase of the task (Figures 2, 3, Supplementary Figure 2). Previous studies of working memory in non-human primates have observed increased power in the gamma frequency band during the delay period in delay-match-to-sample tasks based on averaging data over trials. Here we extended those observations by following the trends in gamma frequency band power as a function of learning over multiple trials (within approximately 30 min to ~1 h) and reported changes in gamma band power as new representations of sequential information are formed.

Increased gamma band activity has been observed during both encoding and memory retrieval in humans (Howard et al., 2003; Sederberg et al., 2007). Previous studies of working memory in humans used a free-recall (Sederberg et al., 2003; Fell et al., 2008) and Sternberg working memory paradigm (Howard et al., 2003) to interrogate the role of gamma oscillations in encoding and retrieval of previously studied items. In the current study learning is defined by performance improvement across trials; we followed the changes in gamma amplitudes over trials. In accordance with previous studies, we noted an increase in gamma band activity in the recall phase with respect to baseline, (Supplementary Figure 3, Figure 2C, Supplementary Figure 2c) but the degree of gamma enhancement was reduced over trials as the subject learned the task (Figures 2, 3, Supplementary Figure 2). We have described this reduction in peak gamma power over trials as a decrease in gamma amplitude.

In addition to learning over trials, other non-specific changes (e.g., attentional variation or fatigue) could take place during the task over the course of a session. Given that attentional modulation has been shown to correlate with changes in gamma power (Fries et al., 2001; Gregoriou et al., 2009), we asked whether the decrease in gamma power could be ascribed to such global non-specific changes. A non-specific interpretation of the findings seems unlikely for several reasons: (i) If the changes were purely driven by decreased attention over the course of trials, we would expect all the sequences to show equal changes in the gamma-band amplitudes. However, the decrease in gamma power was more pronounced for sequences that showed high performance (even though all sequences were randomly interleaved) (Figure 5); (ii) Gamma power was reset across levels (Figure 6); (iii) There was no decrease in gamma power for subjects or sessions where the learning criterion was not reached (Figure 4); (iv) Decrease in gamma power was restricted to the recall phase of the task (whereas one might expect that global factors would affect the baseline and encoding phases as well); (v) Decrease in gamma power was most prominent in the parahippocampal and middle temporal gyri in comparison with other regions that are known to be modulated by attention like the middle frontal and inferior temporal gyri (Supplementary Figure 5, Table 2).

Correct performance in the task involves both recalling the order of the image sequence and pressing the corresponding buttons on the keypad. It is conceivable that the differences between gamma amplitudes in the early and late trials could be modulated by motor improvement rather than sequence learning *per se*. This explanation seems unlikely given that (i) RTs were not correlated with sequence recall performance; (ii) there was no one-to-one correspondence between images in a sequence and buttons on the keypad, and the position of the images on the choice screen were randomized in every trial; (iii) differences were also observed in Task 2 where the motor response and decision were binary; (iv) it seems difficult to explain the sequence dependence in gamma band changes based on descriptions exclusively based on motor improvement; (v) it seems difficult to explain the gamma power reset based on descriptions exclusively based on motor improvement.

The placement of electrodes was governed by clinical criteria, resulting in a broad distribution of electrodes across multiple brain locations (Table 1). As expected, most of the electrodes did not show any significant modulation in gamma-amplitudes with learning. The proportions of learning-modulated electrodes were enhanced in the parahippocampal and middle temporal gyri (Supplementary Figure 5, Table 2). In addition to the well documented link between the hippocampus and memory consolidation (Scoville and Milner, 1957; Squire and Zola-Morgan, 1991), several studies have pointed to a key role for medial temporal lobe structures in declarative learning of sequential information in humans (Reber and Squire, 1998; Hopkins et al., 2004; Holdstock et al., 2005) as well as rats (DeCoteau and Kesner, 2000). Amnesic patients with lesions to the hippocampus and surrounding structures show significant impairment in declarative sequence learning tasks while performance remains intact in implicit or procedural versions of the tasks that do not require attention/consciousness/declarative learning (Nissen and Bullemer, 1987; Willingham et al., 1989). We implemented declarative sequence learning tasks similar to the ones used in earlier studies. The position of the images in the choice screen was randomized in each trial, leading to different motor responses for the same sequences, to ensure that improvement could not be ascribed to pure motor learning.

Several studies have documented effects of adaptation or suppression to repeated presentation of the same stimulus (Li et al., 1993; Gruber et al., 2004; Grill-Spector, 2006). It seems unlikely that our observations can be described as a form of suppression in parallel to the observations in those studies given that (i) there was no consecutive repetition of the same image, (ii) the position of the images in the choice screen was randomized and (iii) the sequences shown in different trials were also randomized (Materials and Methods). Furthermore, we observed no trend in the gamma-band amplitudes in those cases wherein the subject did not learn (Figure 4), and the decrease in gamma band amplitudes correlated with performance for individual sequences (Figure 5). These observations suggest that the modulation effects are sensitive to cognitive demands and behavioral performance rather than purely and passively associated with repetition of visual stimuli.

Earlier events in a series (novel events) typically have a larger influence on behavior and cognition than do subsequent events (Tulving, 2002). This effect of “primacy” is particularly pronounced in the MTL, especially in the parahippocampal and hippocampal regions (Xiang and Brown, 1998; Henson et al., 2003). Neuronal responses to individual images in the MTL have been described in terms of the extent to which the stimuli were novel or familiar to the subject (Li et al., 1993; Stark and Squire, 2000; Brown and Aggleton, 2001; Rutishauser et al., 2006; Viskontas et al., 2006). In particular, single-unit firing in the human hippocampus and parahippocampal cortices increased during presentation of novel stimuli (i.e., was reduced during presentation of familiar stimuli) during a single-image recognition memory task (Rutishauser et al., 2006; Viskontas et al., 2006). In another study modulation of spiking activity was observed with improved learning behavior (Wirth et al., 2003). Neurons in the macaque hippocampus showed changes in stimulus-selective responses with learning in a location-scene learning task. Half of these “changing” neurons showed robust firing in the cue and delay period early in the session, and the firing rates were negatively correlated with learning. The physiological responses described here can be interpreted in terms of increased familiarity with the sequences, which is behaviorally manifested as performance improvement that we refer to as learning. In the context of this task as well as many other tasks examining novelty, learning and familiarity seem to be tautomeric descriptions of the same phenomenon. In contrast with studies of single exposure to individual images and changes that occur after the first exposure, here we described learning/familiarity with image sequences developed over multiple trials and the concomitant physiological changes that directly correlated with behavioral performance.

The biophysics underlying gamma frequency band power changes in IFPs is not clearly understood. Gamma frequency band power from local field potentials (30–100 Hz) has been interpreted to reflect neuronal firing in the vicinity of the electrode (Rasch et al., 2008; Ray et al., 2008; Whittingstall and Logothetis, 2009; Jia et al., 2013). Drawing on these observations, elevated gamma frequency band power may be a useful marker for increased correlated spiking activity; this an assumption that will have to be empirically evaluated). The commitment of new events to memory requires synaptic plasticity and hence tight temporal coordination between neurons (Dan and Poo, 2004). We speculate that modulation in gamma frequency band power may constitute an indirect correlate of plasticity and hence might play an important role in initial learning. According to this interpretation, the decreased gamma frequency band activity after learning and the lack of physiological changes in the absence of learning could constitute a manifestation of the reduced/absent degree of plasticity when there is no behavioral improvement. Another study has observed a time limited role of gamma oscillations in learning suggesting that their function is not in the maintenance of memories (Headley and Weinberger, 2011). While the strength gamma oscillations predicted subsequent memory during initial learning of an auditory fear conditioning task in rats, gamma strength failed to predict further performance once the behavioral asymptote was reached. Taken together, these results highlight the role of changes in gamma oscillations during the early learning and retrieval of memories.

## Grants

This study was supported by a grant from NIH-K08 (William S. Anderson), NIH and NSF (Gabriel Kreiman) and CIMIT (Gabriel Kreiman and William S. Anderson).

## Author contributions

Radhika Madhavan performed experiments, analyzed data and wrote the paper; Hanlin Tang, Daniel Millman, Joseph R. Madsen, and Nathan E. Crone helped perform experiments; Daniel Millman, Radhika Madhavan, William S. Anderson, and Gabriel Kreiman designed the experiment; Fredrick A. Lenz, Travis S. Tierney, Joseph R. Madsen, and William S. Anderson performed the neurosurgical procedures and electrode implantation; William S. Anderson and Gabriel Kreiman supervised the data analyses and wrote the paper. All authors commented and approved the manuscript.

### Conflict of interest statement

The authors declare that the research was conducted in the absence of any commercial or financial relationships that could be construed as a potential conflict of interest.
