# Supplementary material for: Decrease in gamma-band activity tracks sequence learning
Source: Front Syst Neurosci. 2015 Jan 21;8:222. doi: 10.3389/fnsys.2014.00222 (PMC4300908; doi:10.3389/fnsys.2014.00222)

Supplementary Materials (Madhavan et al.)

Supplementary Figure 1: Detailed behavioral performance in epileptic and healthy subjects. (**a1-3, b1-3**) Learning curves for 8 epilepsy subjects (a1-3) and 8 non-epilepsy subjects (b1-3) that participated in Task 1. We defined successful learning as the first point when the subject maintained performance ≥60% during 2 consecutive bins of 10 trials (**Materials and Methods**).
(**a4-6, b4-6**) Distribution of the number of trials to reach learning criterion for epilepsy subjects (a4-6) and non-epilepsy subjects (b4-6) in Task 1. Successful learning for epilepsy subjects was observed in seven sessions (7 subjects). Two sessions (N=2 subjects) did not satisfy the learning criterion ("not learned", gray bar, indicated by arrows in **Fig. 1c**). Seven out of 8 non-epilepsy subjects were able to successfully learn the sequences while one subject was unable to learn the sequences (two sessions on two different days). Here we show behavioral results when considering correct recall of all four images (a1, b1, a4, b4), first image only (a2, b2, a5, b5) and last image only (a3, b3, a6, b6). Error bars show SD.
(**c1-3, d1-3**) Learning curves for 6 epilepsy subjects (c1-3) and 5 non-epilepsy subjects (d1-3) that participated in Task 2. The formats and conventions are the same as in parts a-b.
(**c4-6, d4-6**) Distribution of the number of trials to reach learning criterion for epilepsy subjects (c4-6) and non-epilepsy subjects (d4-6) in Task 2. Successful learning for epilepsy subjects was observed in four subjects. Two subjects did not satisfy the learning criterion ("not learned", gray bar, indicated by arrows in **Fig. 1f**). Four out of 5 non-epilepsy subjects were able to successfully learn the sequences. Here we show behavioral results when considering correct recall of all pairs of images (c1, d1, c4, d4), correct recall in the first question only (c2, d2, c5, d5) and second question only (c3, d3, c6, d6). Error bars show SD.

Supplementary Figure 2: Gamma frequency band power during the recall phase decreased with sequence learning over trials (example from Task 2). Figure format is the same as **Figure 2**. (**a**) Behavioral profile of a representative subject during sequence learning in Task 2 (level 2, 96 trials). (**b**) Associated learning curve. (**c**) Mean gamma frequency band power for "early" and "late" trials for an electrode in the right temporal pole (Talairach coordinates: 28.6, 3.7, -37.0). (**d**) There was a significant reduction in gamma frequency band amplitudes in the late trials compared to the early trials (p=0.009, rank sum test). (**e**) Modulation index (MI) curve (MI: -0.19±0.01 [mean±SEM]).

Supplementary Figure 3: Average gamma frequency band power increased in the recall phase (data averaged over trials). Average power in the gamma frequency band (n=180 trials) for an example electrode on the left temporal pole (Talairach coordinates: -30.2, 13.1, -30.6; inset depicts electrode position). The gray vertical line indicates the presentation of the fixation screen and bars labeled 1-4 represent the four image presentations. The green line represents the onset time for the choice screen (start of the recall phase). The red dashed line depicts the subject's mean response onset time (first

key press). Error bars represent standard error of the mean (SEM) and are shown every 500ms for clarity.

Supplementary Figure 4: Comparison of change in Modulation indices with recall behavior in four frequency bands. Power amplitudes and modulation indices were calculated for four frequency bands from [1-100]Hz. For each frequency band, electrodes that showed significant differences between early and late trials ($p<0.01$, rank sum test) were identified. Average MI as a function of performance for (**a**) [1-10]Hz, (n=51electrodes) (**b**) [10-30]Hz (n=44 electrodes), (**c**) [30-50]Hz, (n=44 electrodes, r = -0.18, $p<1e^{-10}$) and (**d**) [70-100]Hz, (n=61 electrodes, r=-0.15, $p<1e^{-10}$). Frequency bands below 30Hz showed no significant trend of MI with behavior. Figure format is the same as the thick lines in **Figure 3c**. Dotted line indicates MI=0. Note that each line indicates data from both tasks.

Supplementary Figure 5: Location of learning-modulated electrodes
(**a**) The anatomical location of each electrode was obtained by co-registering the MRI and CT scans for each subject (**Material and Methods**). Fifty-one of the 917 electrodes showed significant differences in gamma amplitudes between early and late learning trials. The black circles in this diagram show the proportion of the total number of electrodes in each region that showed modulation between early and late trials. We asked whether the fraction of learning modulated electrodes in each area could be accounted for by the proportion of electrodes in that area. We compared the proportion of electrodes in each region against the null distribution obtained by randomly choosing 51 electrodes from the total of 917 (10,000 iterations). The null distribution for each region is shown as a box plot. Only regions with a total of >10 electrodes were included in this figure. The red line indicates the median of the null distribution and the bottom and top box edges indicate the 25th and 75th percentile. The dashed lines extend to 3 standard deviations from the mean. The numbers of learning modulated electrodes in the PHG (Parahippocampal gyrus) and MTG (Middle temporal gyrus) were over-represented with respect to the null distribution (indicated by asterisk). The location abbreviations and names are shown in **Table 1**. (**b**) Average MI as a function of performance improvement for electrodes in the PHG (n=8, r=-0.61, $p<10^{-5}$) and MTG (n=8, r=-0.46, $p<10^{-5}$). Figure format is the same as the thick lines in **Figure 3c**. Dotted line indicates MI=0.

Supplementary Figure 1:

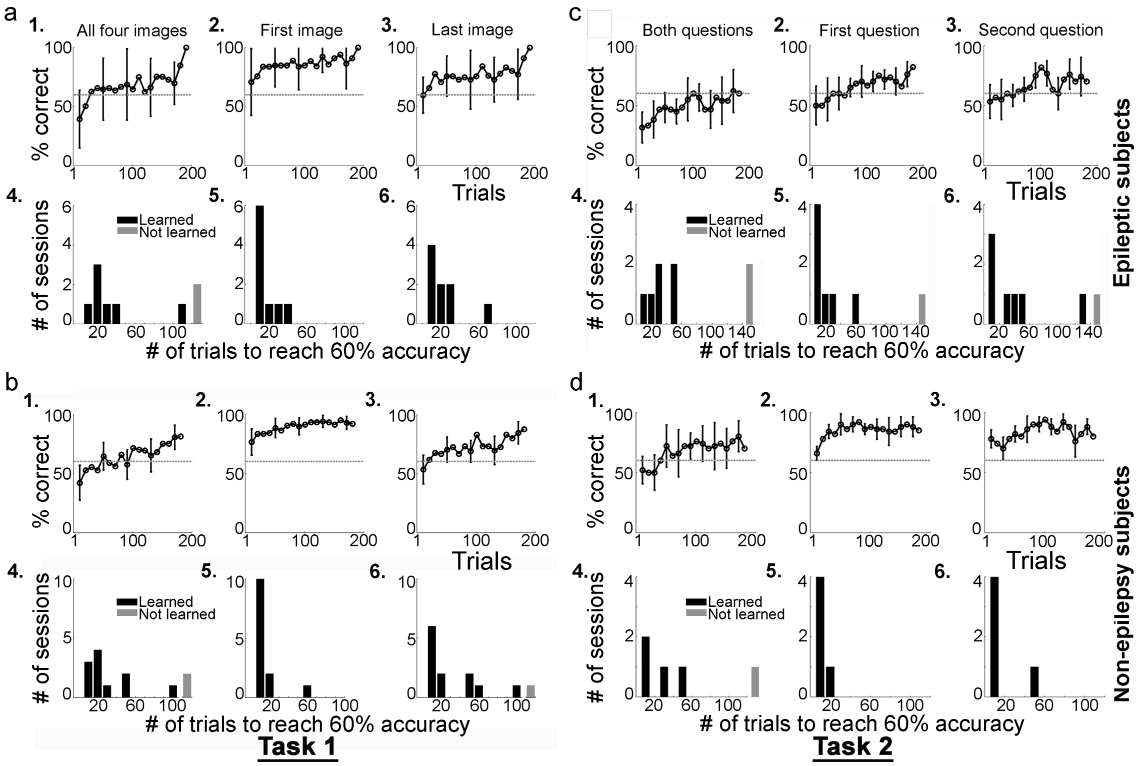

Supplementary Figure 2:

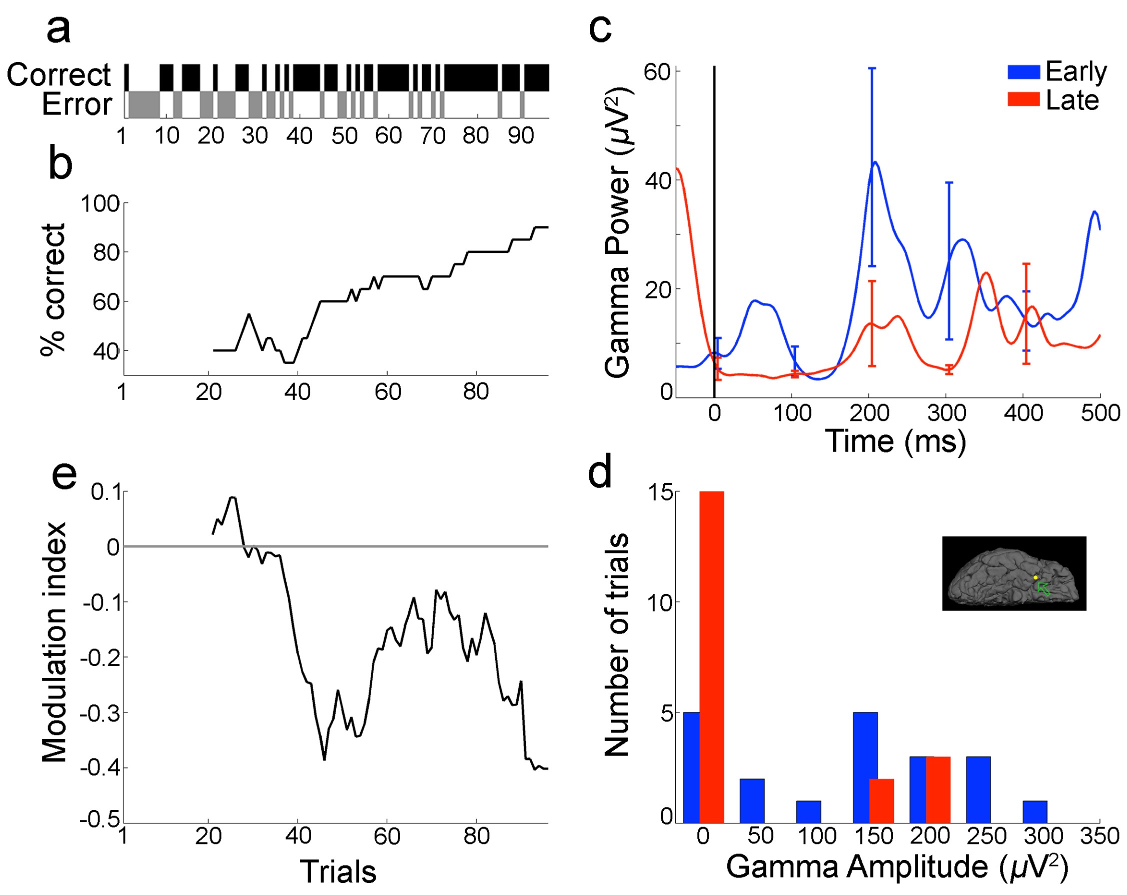

Supplementary Figure 3:

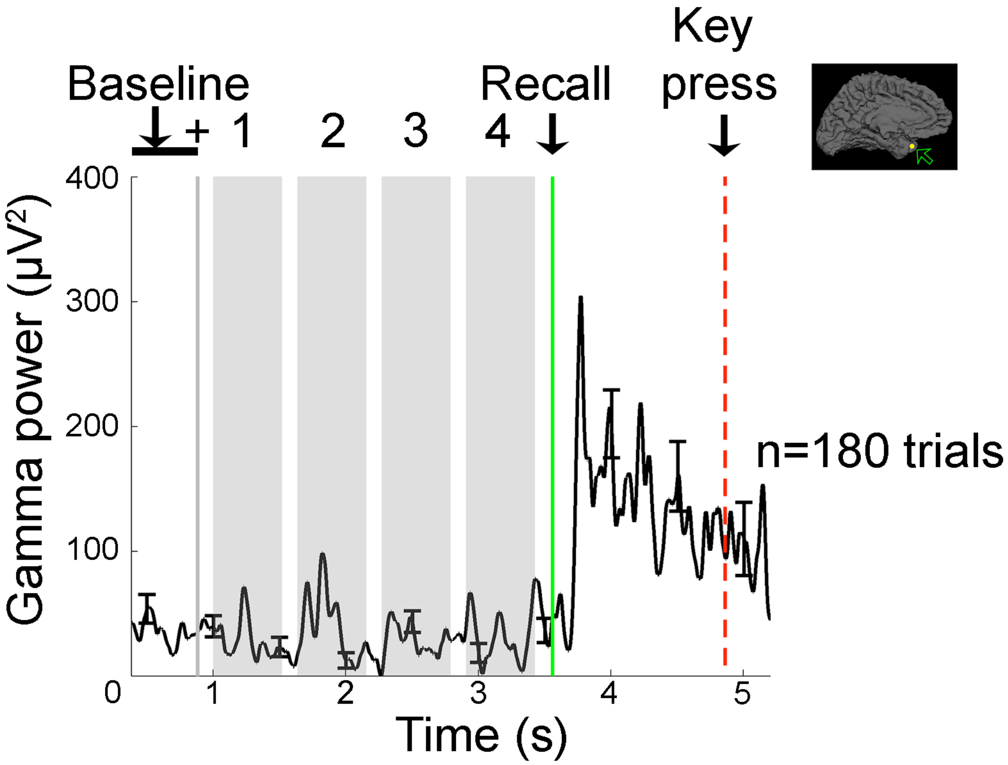

Supplementary Figure 4:

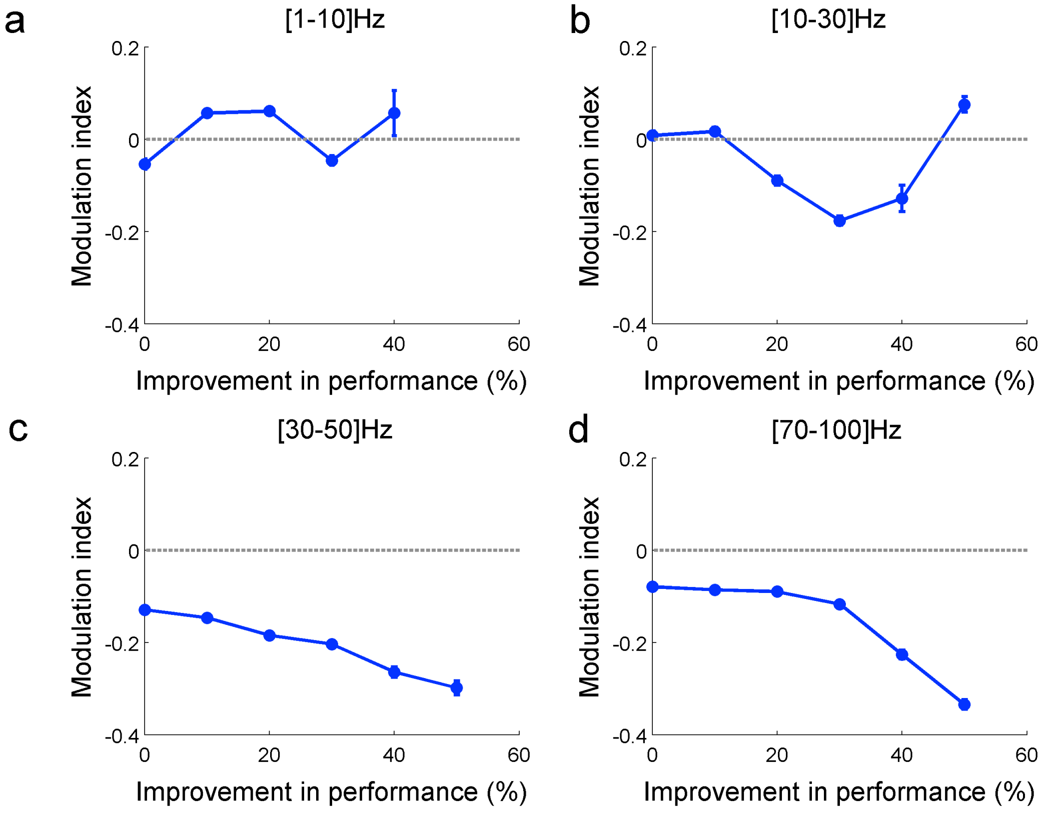

Supplementary Figure 5:

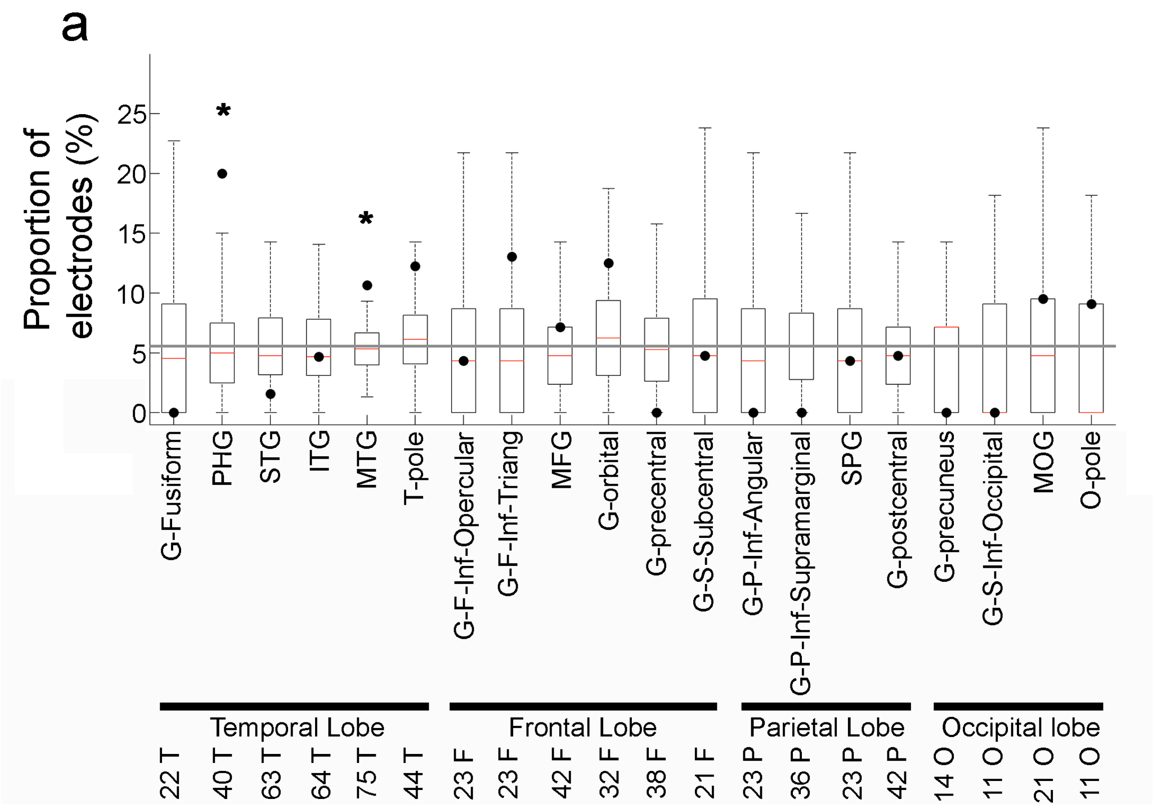

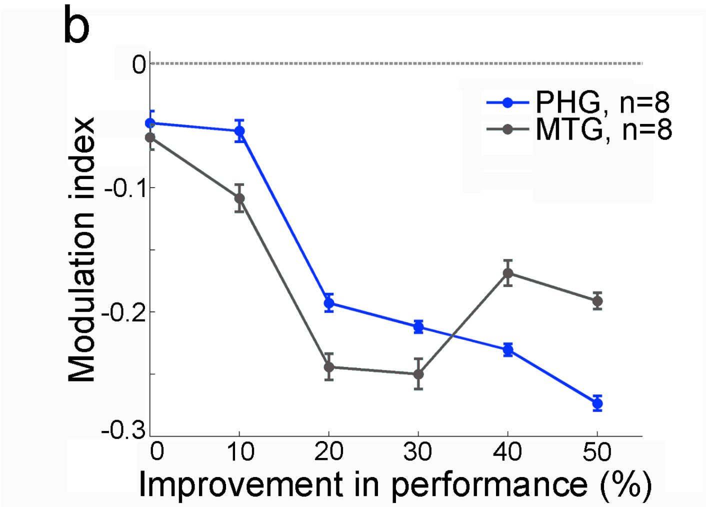

Supplement: Supplementary file 1 [file Presentation1.PDF]
